# miR-96-5p-mediated Inhibition of CD47 contributes to pancreatic tumor regression via activating both innate and adaptive anti-tumor immunity

**DOI:** 10.1186/s12964-025-02582-5

**Published:** 2025-12-05

**Authors:** Shoufang Tong, Shushan Hua, Yunling Wu, Xingxing Xiao, Yeqing Leng, Yubin Wang, Mengfan Sun, Jin Li, Xiping Ou, Wenfeng Zhao, Liping Wang, Yingwei Wang, Shuhua Tan

**Affiliations:** 1 Department of Cell and Molecular Biology, School of Life Science and Technology, State Key Laboratory of Natural Medicines, Jiangsu Key Laboratory of Druhavgg ability of Biopharmaceuticals, China Pharmaceutical University, #639 Longmian Avenue, Jiangning District, Nanjing, 211198 P.R. China; 2 Department of Laboratory Medicine, Tiantai People’s Hospital, Affiliated Hospital of Hangzhou Medical College, Taizhou, China; 3 Center for Laboratory Medicine, Allergy center, Department of Transfusion medicine, Zhejiang Provincial People’s Hospital (Affiliated People’s Hospital), Hangzhou Medical College, Hangzhou, China

**Keywords:** CD47, MiR-96-5p, Tumor immunity, PDAC

## Abstract

Blocking immune checkpoints has become a viable immunotherapy option for cancer. CD47, an anti-phagocytic molecule, engages with SIRPα on macrophages to transmit a “don’t eat me” signal, facilitating immunoevasion. This research examined the molecular pathways influenced by miR-96-5p in pancreatic ductal adenocarcinoma (PDAC) and evaluates its clinical and biological significance. Utilizing bioinformatics, Western blot, luciferase reporter assays and RNA fluorescence in situ hybridization, results revealed that miR-96-5p directly targeted and inversely regulated CD47 expression, indicating patient survival in PDAC. Further investigation into the effects of miR-96-5p on PDAC was conducted through co-culture phagocytosis, antigen presentation, and T cell activation experiments, and mouse PDAC models. The results demonstrated that specifically re-establishing miR-96-5p expression in PDAC cells significantly repressed PDAC tumorigenesis and reprogramed the immunosuppressive microenvironment by diminishing CD47 protein abundance. This modulation encouraged the shift of tumor-associated macrophages (TAMs) into pro-phagocytic M1-like phenotypes via exosomal transfer of miR-96-5p and boosted T cell activation within both peripheral and tumor immune microenvironments. Mechanistically, experiments based on the ovalbumin (OVA) antigen system revealed that the phagocytic function of miR-96-5p enhanced the presentation of tumor antigens to T cells, resulting in more effective activation of antigen-specific T cells, instead of miR-96-5p directly activating T cells. The findings verify that miR-96-5p serves as an effective tumor suppressor, reducing tumor burden by triggering both innate and adaptive anti-tumor immune responses, offering a promising avenue for PDAC immunotherapy improvement.

**Supplementary Information:**

The online version contains supplementary material available at 10.1186/s12964-025-02582-5.

## Introduction

Among all cancers, pancreatic cancer has the worst 5-year survival rate and ranks seventh in cancer-related deaths globally [1, 2]. Pancreatic ductal adenocarcinoma (PDAC), the most prevalent type of pancreatic cancer, remains incurable in 95% of cases. Although surgery combined with chemotherapy offers the best curative potential for PDAC patients, over 80% are diagnosed at advanced stages because of its indolent progression, leading to missed opportunities for early diagnosis and treatment [3]. Despite undergoing a potentially radical resection, the majority of patients experience relapse [4]. Accumulating preclinical data has shown that immunosuppressive effects, including the depletion of cytotoxic T-cell activity within lesions, pose a significant challenge to immunotherapy [5–8]. Therefore, better research into the biology and molecular mechanisms underlying the growth of pancreatic cancer is essential to developing appropriate therapeutic targets.

CD47 is a “marker of self” overexpressed on numerous tumor cell membranes, inhibiting innate immune system attacks on hematologic and solid malignancies [9–11]. The interaction between the SIRPα ligand on myeloid cells, such as macrophages, and the abundant CD47 on tumor cells [12] results in the phosphorylation of SIRPα’s cytoplasmic tail, initiating an inhibitory signaling cascade known as the “don’t eat me” signal [11]. Inhibiting CD47-SIRPα interactions using anti-CD47 or anti-SIRPα antibodies has demonstrated promising therapeutic outcomes for treating blood and solid tumors [13]. Research indicates that antigen-presenting cells (APCs), such as monocytes, dendritic cells, and macrophages, can engulf malignant cells and present tumor antigens to T cells, triggering subsequent adaptive immune responses. Preclinical human tumor models have extensively examined the efficacy of CD47-blocking monoclonal antibodies, which can induce antitumor responses mediated by CD8^+^ T cells [14, 15]. In treating solid tumors, the high molecular weight and immunogenicity of antibodies can lead to patient resistance and inflammation [16, 17], limiting the efficacy of anti-CD47 monotherapy, particularly those with immunosuppressive traits. Consequently, improving strategies focused on the CD47-SIRPα interaction is crucial for advancing cancer treatment.

MicroRNAs (miRNAs) are short RNA molecules, ranging from 19 to 25 nucleotides, that control the expression of protein-coding genes by either destabilizing mRNA or blocking translation [18]. Aberrant miRNA expression patterns have been identified across numerous neoplastic diseases [19, 20], suggesting their role as oncogenes and tumor suppressors by influencing cell proliferation, apoptosis, angiogenesis and therapy resistance in multiple cancer types [20–23]. Emerging data highlights the crucial involvement of miRNA in regulating the tumor immune microenvironment [24, 25]. Furthermore, as miRNAs are antisense nucleotides derived from cells, they demonstrate reduced cytotoxicity and elicit a milder immune response compared to protein, drug, or DNA-based gene therapies [26]. These findings indicate the value of identifying new miRNAs for cancer therapy.

Herein, we reported that miR-96-5p was clinically inversely correlated with CD47 and acted as a tumor-suppressive miRNA in PDAC. Data verified that restoration of miR-96-5p in PDAC cells significantly inhibited PDAC malignant phenotypes. Mechanism investigations revealed that miR-96-5p suppressed CD47 expression by acting on the 3’-UTR (“UGCCAA”) of CD47 mRNA and encouraged macrophages to polarize to the M1 type via exosome transfer, leading to the phagocytosis and direct elimination of PDAC cancer cells. Additionally, miR-96-5p-mediated phagocytosis be indicated to trigger a strong anti-tumor T-cell response to indirectly eradicate malignant tumors. Therefore, our findings highlight miR-96-5p as a crucial tumor suppressor in the progression of PDAC, with biological, mechanistic and clinical effects on human PDAC and the CD47/SIRPα signaling pathway.

## Materials and methods

### Reagents

Opti-MEM (Cat. no. 22600050), RPMI-1640 (Cat. no. 31800022), and DMEM (Cat. no. 12800017) were sourced from Thermo Fisher Scientific, Waltham, MA, USA. CFSE (Cat. no. 22022) was purchased from AAT Bioquest, California, USA. eFluor™ 670 (Cat. no.65–0840−85), Lipofectamine 3000™ (Cat. no. L3000015), and BbsI (Cat. no. ER1012) were obtained from Invitrogen, Carlsbad, California, USA. M-CSF was purchased from Peprotech (California, Carlsbad, USA). RNAiso Plus (Cat. no. 9109), SacI (Cat. no. 1627), XhoI (Cat. no. 1094 S), XbaI (Cat. no. 1634), and BamHI (Cat. no. 1010 S) were sourced from TaKaRa, Dalian, China. The anti-CD47 antibody (Cat. no. ab175388) was sourced from Abcam, Cambridge, UK. Phorbol 12-myristate 13-acetate (PMA, Cat. no. S1819) and luciferase reporter gene assay kit (Cat. no. RG088S) were obtained from Beyotime, Shanghai, China. Anti-SIINFEK6/H-2Kb-APC (Cat. no. 17–5743−80), anti-CD3-PerCP-cy5.5 (Cat. no. class="convertEndash" ID="EN4">45-0031 class="convertMinus" ID="MN3">−80), anti-CD44-FITC (Cat. no. MA1-81257), anti-MHC-II-PE (Cat. no. 12–5320−80), and anti-CD47 blocking (Clone miap-301, Cat. no. 16–0471−81) were from eBioscience. FACS antibodies were procured from BioLegend (San Francisco, USA): anti-F4/80-Alexa Fluor^®^647 (Cat. no. 157314), anti-F4/80-APC (Cat. no. 123121), anti-F4/80-PE (Cat. no. 111704), anti-CD11b-PercP5.5 (Cat. no. 101227), anti-CD206-APC (Cat. no. 141707), anti-CD8α-APC (Cat. no. 100711), anti-CD4-APC (Cat. no. 100411), anti-NK1.1-APC (Cat. no. 156505), and anti-CD62L-PE (Cat. no. 104407).

### Bioinformatics analysis

We used different miRNA target-predicting algorithms (starBase v2.0 (http://starbase.sysu.edu.cn/starbase2/index.php) [27], TargetScan (http://www.Targetscan.org/vert_72) [28], DIANA-microT (http://www.microrna.gr/microT-CDS) [29], PicTar (https://pictar.mdc-berlin.de/) [30]) to predict potential CD47-targeting miRNAs. Microarray data and patient information were obtained from TCGA databases and the GEO repository (accession numbers: GSE71533, GSE62452, GSE60978). CD47 expression levels in various tumor and normal samples were analyzed using the Gene Expression Profiling Interactive Analysis (GEPIA, http://gepia.cancer-pku.cn/) [31] tool to access the TCGA databases. Survival analysis of CD47 and miR-96-5p using LinkedOmics (https://www.linkedomics.org/login.php) [32]. The DIANA-miRPath v3.0 was used to perform a Gene Ontology (GO) enrichment analysis based on public TarBase and microT-CDS datasets, focusing on biological process pathways potentially altered by miR-96-5p [33].

### Ethics approval

Human tissue experiments adhered to the Helsinki criteria and were approved by the Tiantai People’s Hospital of Zhejiang Province (Approval no.: QT2025023-01). Thirty cases of pathological paraffin tissues with PDAC were collected by the Tiantai People’s Hospital of Zhejiang Province. Because the study was retrospective, the requirement for informed consent was waived. All animal experiments adhered to the regulations set by the Animal Ethics Committees of China Pharmaceutical University and the National Institutes of Health’s Guide for the Care and Use of Laboratory Animals (Approval no.: 2022-02−022).

### Mice

Male mice (6–8 weeks) and female OT-I and OT-II mice (6 weeks) in a C57BL/6 background were obtained from Qinglongshan Experimental Animal Breeding Farm. Both OT-I and OT-II mice carry transgenic Vα2 Vβ5 TCRs. CD8^+^ T cells in OT-I mice possess transgenic TCRs that specifically target the OVA_257 − 264_ peptide presented by H2-kb. OT-II mice possess CD4^+^ T cells with transgenic TCRs that specifically recognize the OVA_323 − 339_ peptide presented by IAb. These mice were housed in a specific pathogen-free and temperature-controlled room environment at the China Pharmaceutical University Animal Center.

### Cell culture

Human PDAC (PANC-1, AsPC-1, BxPC-3, MIA PaCa-2), leukemia (THP-1), and embryonic kidney (HEK293T) cell lines were obtained from the Chinese Academy of Sciences’ cell bank in Shanghai, China. The Panc02 and HPDE6-C7 cell lines were sourced from the American Type Culture Collection. Cells were cultured at 37 °C in a humidified 5% CO_2_ atmosphere using DMEM or RPMI-1640 (AsPC-1, BxPC-3, and THP-1) media supplemented with 10% fetal bovine serum, 100 units/mL penicillin, and 100 µg/mL streptomycin.

### Cell transfection

Synthetic miRNA duplexes and inhibitors (Table S1) were purchased from GenePharma (Shanghai, China). For transfection of miR-96-5p mimics (miR-96-5p, 5′-UUUGGCACUAGCACAUUUUUGCU-3′) and inhibitors (anti-miR-96-5p, 5′- AGCAAAAAUGUGCUAGUGCCAAA-3′), PDAC cells were planted 24 h earlier to reach a growth density of about 70%. Transient transfection was conducted with Lipofectamine 3000TM following the manufacturer’s guidelines. The control mimic (5′-UUCUCCGAACGUGUCACGUTT-3′) and control inhibitor (5′-CAGUACUUUUGUGUAGUACAA-3′) served as controls for miR-96-5p mimic and inhibitor transfections, respectively. Within the predetermined time after transfection, the cells were collected and further functional experimental tests were conducted.

### qPCR analysis

Total RNA was isolated utilizing RNAiso Plus reagent (Takara). mRNA levels were quantified by synthesizing complementary DNA (cDNA) with the cDNA Synthesis Kit (Beyotime). miRNA quantification was conducted using reverse transcription with the miRNA cDNA Synthesis Kit (Sangon Biotech). The reaction solution was prepared using SYBR Green Mix (Beyotime) and quantified analyses were performed on an MX3000P™ qPCR instrument. Using the 2^−ΔΔCt^ algorithm, gene expression was normalized against GAPDH or U6. Table S2 provides details of the primers synthesized by Sangon Biotech.

### Western blot analysis

The total protein was extracted using RIPA with 1mM PMSF added. After protein quantification, it was separated on 10% SDS-PAGE electrophoresis gel. Protein blots were transferred to 0.22 μm PVDF membranes (MilliporeSigma, USA) and blocked with 5% (w/v) nonfat milk for 2 h at room temperature. Blots were exposed to primary antibodies CD47 (1:1000) and GAPDH (1:2000, Cat. no. D110016, Sangon Biotech) overnight at 4 °C, and then incubated for an hour at room temperature with an HRP-conjugated goat anti-rabbit IgG secondary antibody (1:5000, Cat. no. D110058, Sangon Biotech). Enhanced electrochemiluminescence (ECL) fluid was employed to visualize protein bands, and ImageJ software was used for their quantification.

### Luciferase reporter assay

The 3′-UTR regions of human CD47 (NM_001777), which include potential miR-96-5p binding sites, were amplified using specific primers (Table S3) and cloned into the pmirGLO vector (Promega) at the SacI and XhoI restriction sites. Site-directed mutagenesis was employed to generate mutant vectors by targeting the predicted miR-96-5p binding site within the 3′-UTR of the CD47 sequence. All constructs were verified through Sanger sequencing by Sangon and then co-transfected with miR-96-5p mimics or inhibitors into HEK293T cells for 24 h. The Luciferase Reporter Gene Assay Kit (Beyotime) was used to measure the activities of Firefly and Renilla luciferases.

### CRISPR/Cas9-mediated miR-96-5p knockout

sgRNAs targeting the flanks of human or mouse pre-miR-96 were designed using online CRISPR software (http://crispr.mit.edu/) (Table S4) and cloned into the pX459 vector to create recombinant plasmids for gene knockout. Recombinant plasmids targeting both sides of pre-miR-96 were used to co-transfect PDAC cells. PDAC cells are exposed to 2 µg/mL puromycin for 48 h following a 24-hour period after transfection to eliminate non-infected cells. Then, monoclonal cells were isolated by adjusting the cell solution to 10 cells/mL and dispensing 100 µL into each well of a 96-well plate. After allowing the cells to remain undisturbed for 1–2 weeks, visible colonies were transferred to new wells using a sterile pipette tip for further amplification. PCR and sequencing of PCR products were conducted to identify successfully knocked-out clones (miR-96-5p^−/−^). Finally, qPCR analysis further verified miR-96-5p′s expression loss.

### Phagocytosis assay

Human macrophages were derived from THP-1 monocytes by treating them with 100 nM PMA for three days. Macrophages derived from murine bone marrow (BMDMs) were collected by removing the femur and tibia from C57BL/6 mice and extracting the marrow cells using RPMI 1640 medium without serum. Then marrow cells were differentiated into macrophage-like cells by being cultured in RPMI-1640 complete medium containing 10 ng/mL M-CSF for 5 days. Before conducting phagocytosis assays, human THP-1-derived mature macrophages (labeled with eFluor™670) and murine BMDMs were cultured without serum for 2 h, followed by a 3–4 h co-incubation with CFSE-labeled tumor cells, including those transfected with miRNA mimics/inhibitors or miR-96-5p^−/−^ cells. Cells were collected (BMDMs were labeled with F4/80-Alexa) and suspended in 400 µL cold PBS. Phagocytosis was assessed using fluorescence-activated cell sorter (FACS), and the proportion of CFSE-labeled cells within eFluor™ 670^+^ or F4/80^+^ phagocyte populations was determined with FlowJo software 7.6 (FlowJo, Oregon, USA).

### Cell proliferation, migration, and cell cycle assay

Cells were inoculated at a density of 3 × 10³ per well in a 96-well plate for the proliferation assay, followed by transfection with miRNA mimics or inhibitors. MTT (10 µL) was added 4 h post-transfection at 0, 24, 48, and 72 h, maintaining a temperature of 37 °C. After carefully removing the culture medium, dissolve formazan with dimethyl sulfoxide (DMSO) and measure the absorbance at 570 nm.

For cell migration assay, PDAC cells were implanted in 6-well plates until cell confluency reached 70%. 200 µL pipette tips scratched the cell monolayer, which was subsequently washed twice with PBS and transfected with miRNA mimics or inhibitors. Then, serum-free DMEM medium was added, and the cells were incubated for another 24 h. Using an inverted microscope (Zeiss, Oberkochen, Germany), the wound gaps were documented at the time points of 0 and 24 h post-transfection. The gap width was measured using ImageJ, and the wound closure rate was calculated with the formula: wound closure rate = [(A-B)/A] × 100%. Where A represents the initial wound width and B denotes the wound width after 24 h.

For cell cycle analysis, 5 × 10^5^ cells were cultured in 6-well plates and transfected with miRNA mimics or inhibitors for 48 h. Two hours before harvesting, the cells were treated with 10 µM EdU (5-ethynyl-2′-deoxyuridine). During DNA replication, EdU is integrated into newly synthesized DNA and labeled with an azide-coupled fluorophore (Alexa 488), while the total DNA content is stained with the red fluorescent dye PI (Beyotime). Following the manufacturer’s instructions, we employed the Click-iT EdU Assay Kit (Beyotime) to evaluate EdU incorporation and measure DNA content. FACS was utilized for cell cycle analysis, and distribution was assessed using FlowJo software version 7.6.

### Exosome isolation

After 72 h of cell cultivation, debris is eliminated by centrifuging the cell supernatant at 300 × g for 10 min. The supernatant is then extracted by centrifuging it for another 10 min at 2000 × g at 4 °C. Centrifuge the large vesicles at 10,000 × g for 30 min. For one hour, ultracentrifuge the supernatant at 110,000 × g while retaining the pellet that contained the exosomes. The exosome precipitates underwent further centrifugation after being cleaned with PBS. Exosomes were imaged with a transmission electron microscope (Thermo Scientific, USA) following placement on copper mesh and negative staining with 2% phosphotungstic acid.

### Antigen presentation assay

Murine Panc02 cells expressing cOVA were transfected with miR-96-5p mimics or inhibitors for 48 h, with or without 10 µg/mL anti-CD47 mAb (eBioscience) blocking, and subsequently co-cultured with BMDMs pre-starved for 2 h for an additional 24 h. Cells were collected on ice and washed twice with cold PBS. The OVA_257− 264_ peptide presented on MHC-I on the BMDM surface was identified using APC-labeled anti-SIINFEK6/H-2Kb (eBioscience), an antibody specific to the OVA peptide SIINFEKL (OVA_257− 264_) bound to H-2Kb, by staining on ice for 20 min. Antigen presentation was evaluated using Guava EasyCyte™ Flow Cytometry determined by the percentage of APC^+^ cells among F4/80^+^ phagocytes.

### T-cell isolation

Under sterile conditions, spleens from 6- to 12-week-old OT-I or OT-II mice were excised and rinsed three times using chilled PBS. The spleen was mechanically disrupted using a syringe latex head in PBS with 2% FBS and passed through a strainer with a 70 μm pore size. Erythrocytes were removed using ACK lysis (5 mL per spleen) (Beyotime). Naïve T cell isolation kits (STEMCELL) weere used to isolate Naïve CD8^+^ and CD4^+^ T cells.

### T-cell priming assay

Panc02-cOVA cells and BMDMs were treated and co-cultured as described in the murine antigen presentation assay. Naïve T cells, sorted magnetically and labeled with CFSE (Beyotime), were added to cocultures for 72 h to analyze T cell proliferation. FACS was used to analyze CFSE dye dilution in CD4^+^T (CD3^+^ CD4^+^) and CD8^+^T (CD3^+^ CD8^+^) populations after staining cells with CD3, CD4, and CD8 (BioLegend). Naïve T cells were introduced to cocultures for 72 h to facilitate T-cell maturation. Subsequently, cells were stained with antibodies targeting CD3, CD4 or CD8, CD44 (Clone >5035-41.1D; eBioscience), and CD62L (Clone MEL-14; BioLegend). FACS was employed to evaluate T cell maturation, identifying naïve T cells as CD44^−^ CD62L^+^, central memory T cells as CD44^+^ CD62L^+^, and effector memory T cells as CD44^+^ CD62L^−^.

### Lentiviral constructs and infection

To achieve miR-96-5p overexpression in Panc02 cells, the mmu-pri-miR-96 (Gene ID 723886) was amplified with designated primers (Table S2) and inserted into the pCDH-MSCV-MCS-EF1-copGFP-T2A-Puro lentivirus vector (Lv-miR-96) at the XbaI and BamHI restriction sites. HEK293T cells were co-transfected with either the recombinant plasmid or an empty vector, alongside packaging vectors psPAX2 (Addgene) and pMD2.G (Addgene), utilizing Lipofectamine 3000™ reagent. Then, with the assistance of adding 8 µg/mL polystyrene, Panc02 cells were infected with the generated lentivirus. Monoclonal isolation, FACS screening, and qPCR aassays were further conducted to screen the clones with high expression of miR-96-5p.

### Subcutaneous model of PDAC

A subcutaneous tumor model was developed by injecting Panc02 cells (Panc02-wt, -miR-96-5p^−/−^, -Lv-vector and -Lv-miR-96). Cells were prepared at a concentration of 1 × 10^6^ cells/mL in 1 mL of pre-cooled PBS, kept on ice, and 100 µL of this suspension was subcutaneously injected into the right flank of the mice. Tumor size was assessed on day 6 post-injection and subsequently every two days, with tumor volume calculated using the formula V = 0.5 × L (length) × W² (width). On day 27, mice were euthanized to obtain peripheral blood, tumors, and spleens for immune cell subset examination and histological analysis.

### Orthotopic model of PDAC

To establish an orthotopic PDAC model, the left upper abdomen of anesthetized mice was disinfected with iodophor, and the skin and peritoneum were incised about 1 cm to expose the spleen and pancreas. Using blunt tweezers, the pancreas tail was gently elevated for full exposure, allowing for the slow injection of 4 × 10^4^ Panc02-Luc cells in 50 µL PBS into the pancreatic parenchyma with a 1 mL insulin syringe. The spleen and pancreas were repositioned, and the skin and peritoneum were sutured with 3 − 0 Vicryl. Following Chen et al. [34], an adeno-associated virus (AAV) control and an AAV vector expressing miR-96-5p were developed and packaged by Obio Technology Co., LTD (Shanghai). AAV2 genomes were packaged within Y447F and Y773F mutant AAV8 capsids to form a viral vector with high efficiency for pancreatic transduction (AAV-PAN). On the second day following the implantation, 100 µL PBS or AAV (AAV-vector and AAV-miR-96; 2.5 × 10^11^ vg/animal) was intraperitoneally administered. Notably, since the wound has not yet fully healed, the mice should be handled with care to prevent any strain on the wound. Two weeks after the initial intraperitoneal (i.p.) administration, a second i.p. injection was administered. Ten days after the tumor cell transplant, mice were imaged with a small animal in vivo imaging system (IVIS): the mice were intraperitoneally administered D-luciferin potassium salt (Beyotime) with a dosage of 10 µL/g based on their weight, and after 10 min, the mice were imaged and fluorescence values were calculated. After 30 days, all experimental mice were euthanized to excise tissues for further analysis.

### Isolation of monocytes and FACS analysis of immunocyte subsets

Tumor masses were sectioned into 1–3 mm³ pieces and digested at 37 °C for 1 h using a solution with 150 µg/mL DNase I, 150 µg/mL hyaluronidase, and 200 U/mL collagenase type I. After passing dissociated cells through a 70-µm strainer to discard undigested tumor fragments, monocytes were isolated using a Mononuclear Cell Separation Kit (Solarbio). Mononuclear cells were isolated from spleens by washing the spleens with sterile, pre-cooled PBS and filtering them through a 70-µm mesh. The erythrocytes in the spleen were then lysed with ACK lysis buffer (Beyotime). Mononuclear cells were isolated from mouse peripheral blood utilizing a Solarbio Peripheral Blood Isolation Kit and density gradient centrifugation. The obtained cells were stained with anti-mouse antibodies to identify specific immune cell populations: M1 macrophages (CD11b^+^ MHC-Ⅱ^+^ F4/80^+^), M2 macrophages (CD11b^+^ F4/80^+^ CD206^+^), CD4^+^ T cells (CD3^+^ CD4^+^), CD8 + T cells (CD3^+^ CD8^+^), and natural killer (NK) cells (CD3^+^ NK1.1^+^).

### Histological evaluation

Embedded in paraffin, tumors were cut into slices measuring 4–5 μm and affixed to glass slides. Paraffin sections underwent dewaxing in xylene, rehydration in ethanol, and antigen retrieval by heating in a citrate buffer (pH 6.1) at 95 °C. Slides were treated with 3% H_2_O_2_ to inhibit endogenous peroxidase, then blocked with 10% goat serum and incubated with an anti-CD47 antibody, followed by a biotinylated secondary antibody. The slides underwent color development with the addition of DAB solution (Sangon Biotech). Tumor sections were stained with hematoxylin and eosin (H&E). To assess the toxicity and targeting of AAV-PAN, major organs were collected, embedded in OCT freezing medium, and stained with either H&E or DAPI. Then, morphological changes and GFP were observed under microscopy (Zeiss, Oberkochen, Germany).

### RNA fluorescence in situ hybridization (FISH) analysis

For miR-96-5p, GenePharma designed and synthesized Cy3-labeled targeted probes. Signals are identified using the FISH kit (GenePharma, China). Tissue sections of patients with pancreatic cancer were treated with protease K at 37 °C for 20 min. After pre-hybridization, the slices were hybridized overnight using the miR-96-5p FISH probe (AGCAAAAATGTGCTAGTGCCAAA) at 37 °C. Each slide was soaked in 100 µL of hybridization post-treatment solution for 15 min and then washed with PBS once. The cell nucleus was exposed to the DAPI staining solution for 2 min. Confocal images were acquired under a microscope.

### Statistical analysis

At least three repetitions were done for each experiment, and the data is reported as means ± SEM (*n* ≥ 3). An unpaired Student’s t-test was used for statistical analysis between two groups, while a one-way ANOVA was applied for comparisons among multiple groups. A two-way ANOVA was conducted to analyze the primary effects on cell proliferation and tumor growth curves. Pearson’s correlation analysis assessed the clinical relationship between CD47 and miR-96-5p. Statistical significance was defined as a *P*-value less than 0.05.

## Results

### CD47 is aberrantly upregulated in PDAC patients

**Fig. 1:**
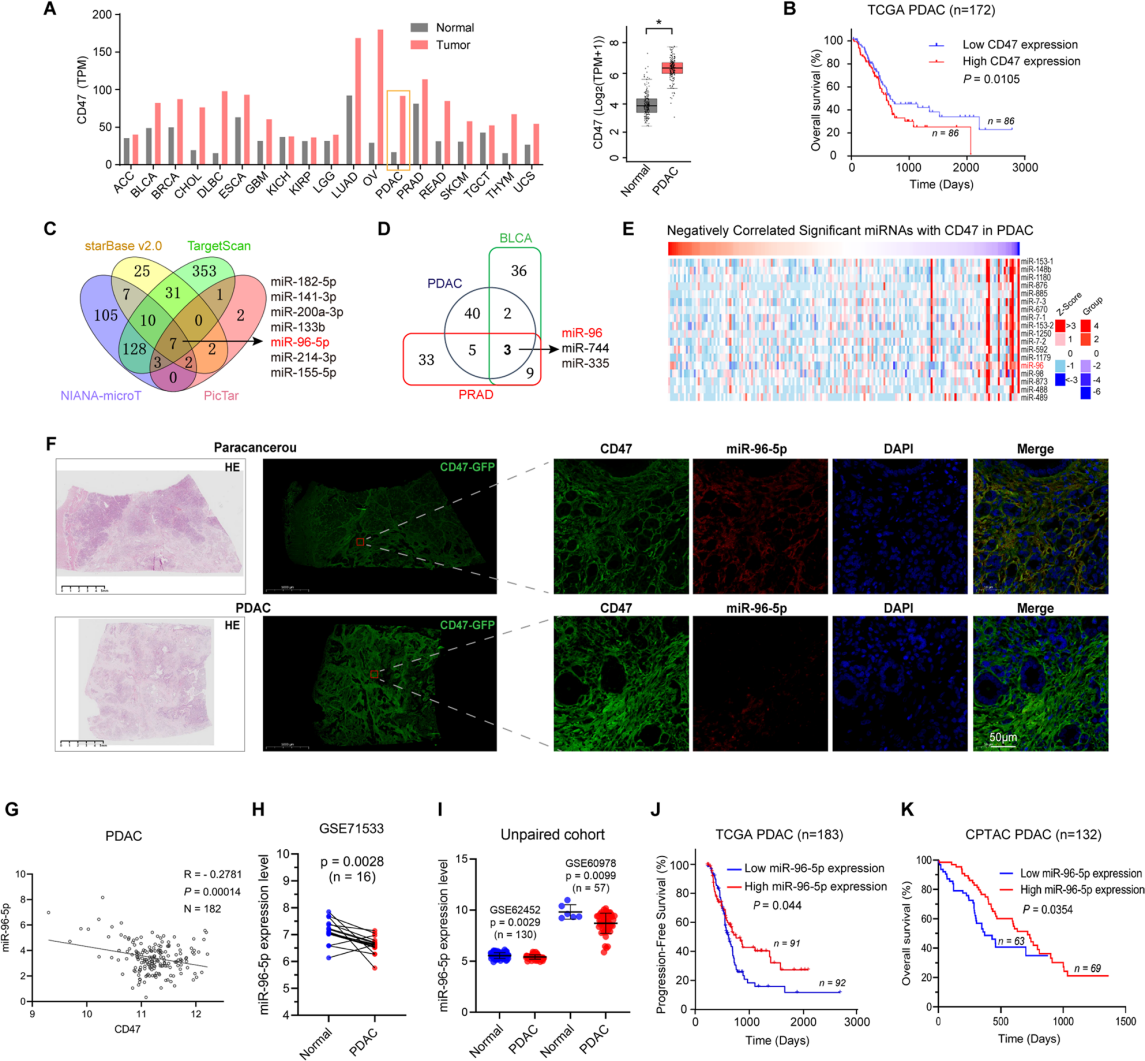
miR-96-5p shows an inverse relationship with CD47 expression, and higher levels of miR-96-5p are associated with favorable prognosis in PDAC patients. **A** Comparison of CD47 expression from GEPIA in a variety of tumor tissues (red) along with their corresponding matched normal tissues (grey). Right: 171 normal pancreas and 179 pancreatic carcinoma tissues were compared for CD47 expression. **B** The Cox regression curves for the survival analysis of 172 PDAC patients who were classified as either high (red) or low (blue) in CD47 expression. **C** Venn diagram showing the overlap of potential miRNAs targeting CD47. **D** MiRNAs identified by LinkedOmics exhibited a statistically significant inverse correlation with CD47 in PDAC, PRAD, and BLCA. **E** A heat map displaying the miRNAs that exhibited an inverse correlation with CD47 in PDAC. **F** Images of HE and IF on the sections revealed the expression of CD47 and miR-96-5p in the adjacent and PDAC tissues. **G** A correlation analysis between miR-96-5p and CD47 was conducted in PDAC (*n* = 182) using data from TCGA. **H** Analysis of miR-96-5p levels in PDAC versus normal pancreatic tissues using data from the GEO database (GSE71533). **I** Comparison of miR-96-5p levels in PDAC and normal tissue samples was conducted using two independent GEO datasets: GSE62452 and GSE60987. **J-K **Progression-Free Survival(TCGA, J)and Overall Survival (CPTAC, K) analysis identified correlations between miR-96-5p and survival probability in PDAC

CD47, a key cell surface receptor, exhibits significant overexpression in tumor tissues, including PDAC, compared to non-tumor tissues, as revealed by TCGA database analysis using GEPIA (Fig. 1 A). Utilizing LinkedOmics to categorize PDAC patients into “CD47 high” and “CD47 low” group, Cox regression analysis revealed that elevated CD47 expression correlates with decreased survival probability in PDAC (Fig. 1B).

### miR-96-5p downregulation is closely associated with low survival rate in PDAC patients

We rationally identified miRNAs targeting the immune checkpoint CD47 gene through comprehensive bioinformatics method: (i) utilizing different validated miRNA databases and predictive tools for miRNA-gene identification; (ii) conducting differential expression analysis; (iii) examining correlations with patient outcomes; and (iv) assessing expression correlations between candidate miRNAs and their predicted targets. Based on 3′-UTR binding sites, miRNA target-predicting algorithms (starBase v2.0, TargetScan, DIANA-microT, and PicTar) consistently identified seven potential miRNAs: miR-182-5p, miR-141-3p, miR-200a-3p, miR-133b, miR-96-5p, miR-214-3p, and miR-155-5p (Fig. 1 C). Then, using LinkedOmics, we identified miRNAs in PDAC, PRAD, and BLCA that had a substantial negative correlation with CD47 expression. We discovered that the three malignancies shared three potential miRNAs: miR-96, miR-744, and miR-335 (Fig. 1D). In PDAC, miR-96 was the thirteenth miRNA with the strongest negative correlation to CD47 expression, as indicated by the heat map (Fig. 1E). To clarify the clinical correlation of miR-96-5p and CD47 protein abundance, we performed immunofluorescence staining on 26 cases of PDAC tissues and adjacent tissues using anti-CD47 and miR-96-5p probes, respectively. Compared with the adjacent tissues, PDAC tissues had higher CD47 expression but decreased miR-96-5p expression (Fig. 1 F). Fig. 1G and Fig. S1A-B illustrate an inverse correlation between miR-96-5p and CD47 across 182 PDAC, 543 PRAD, and 423 BLCA samples. Additionally, radiation therapy notably elevated miR-96-5p levels in PDAC patients (Fig. S1C). Analysis of public GEO datasets revealed consistent downregulation of miR-96-5p in PDAC tissues compared to nontumor tissues across one paired and two nonpaired PDAC cohorts (Fig. 1H and I). Moreover, reduced miR-96-5p expression correlated with lower progression-free survival and overall survival in PDAC (Fig. 1 J and K). The results suggest that miR-96-5p is pivotal in the development and progression of PDAC.

### miR-96-5p is down-regulated in multiple PDAC cells and directly targets CD47

**Fig. 2:**
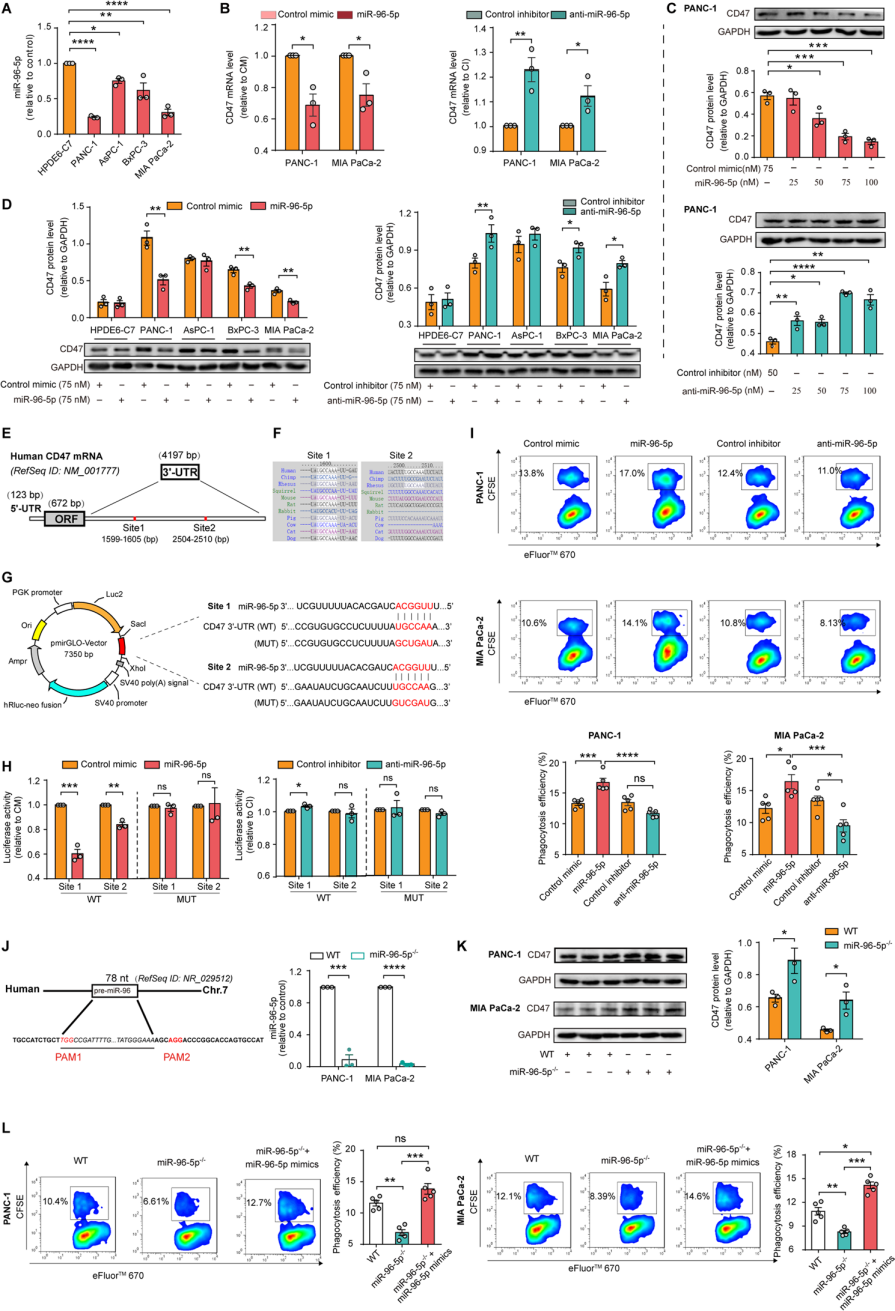
MiR-96-5p directly targets CD47 to facilitate phagocytosis of PDAC cells by macrophages. **A** qPCR analysis of miR-96-5p levels in a normal pancreatic cell line and various PDAC cell lines (*n* = 3). **B ** qPCR analysis of CD47 mRNA levels post-transfection with miR-96-5p mimics and inhibitors (*n* = 3). **C** Western blot analysis of the CD47 protein levels post-transfection with varying concentrations of miRNAs (*n* = 3). **D** Western blot analysis of the CD47 protein levels in a normal pancreas cell line or multiple PADC cell lines (*n* = 3). **E** Diagram depicting the 3′-UTR of CD47 mRNA with two potential miR-96-5p binding sites. **F** TargetScan 7.2 showed the conservation of the seed sequence “UGCCAA” for miR-96-5p targeting in human CD47 3′-UTR across multiple species. **G** Subcloning wild-type (WT) or mutant (MUT) 3′-UTR segments into the *SacI* and *XhoI* sites of the pmirGLO plasmid. The seed sequences were highlighted in red. **H** Luciferase reporter assays were performed to examine the binding of miR-96-5p with the CD47 3’-UTR (*n* = 3). **I** The engulfment of PDAC cells by THP1-derived macrophages was examined using FACS (*n* = 5). **J** A diagram illustrating the deletion of pre-miR-96 mediated by the CRISPR/Cas9 system. Pre-miR-96 and PAM sequences are denoted by underlining and red highlighting, respectively. Right: qPCR analysis of miR-96-5p expression in both wild-type and miR-96-5p^−/−^ PDAC cells. **K** Western blot analysis of CD47 expression in wild-type and miR-96-5p^−/−^ PDAC cells (*n* = 3). **L** Analysis of phagocytosis in both wild-type and miR-96-5p^−/−^ PDAC cells by THP-1-derived macrophages, with or without miR-96-5p overexpression (*n* = 5)

Figure 1 K and L illustrate that miR-96-5p expression is lower in PDAC tissues compared to adjacent normal tissues, according to GEO datasets. Besides, RT-qPCR analysis revealed that miR-96-5p expression was significantly lower in four PDAC cell lines compared to the normal HPDE6-C7 cell line (Fig. 2A). To determine how miR-96-5p regulates CD47 abundance, qPCR and Western blot analysis was conducted. Results showed that miR-96-5p mimics dramatically decreased, while anti-miR-96-5p increased CD47 mRNA expression (Fig. 2B). Besides, 48 h post-transfection with varying concentrations of miR-96-5p or anti-miR-96-5p, it was observed that miR-96-5p dose-dependently reduced, whereas anti-miR-96-5p increased CD47 protein expression (Fig. 2C). We further evaluated the protein-level changes of CD47 induced by miR-96-5p in normal pancreatic and various PDAC cells to verify its regulatory role. As shown in Fig. 2D, CD47 expression was lower in the normal HPDE6-C7 cell line compared with four PDAC cell lines. In PDAC cell lines PANC-1, BxPC-3, and MIA PaCa-2, miR-96-5p significantly decreased, while anti-miR-96-5p elevated CD47 expression. We subsequently demonstrated that miR-96-5p’s inability to reduce CD47 protein levels in HPDE6-C7 and AsPC-1 cells might result from low transfection efficiency (Fig. S2).

A luciferase vector containing CD47 mRNA 3′-UTR (RefSeq ID NM_001777) was used in a dual-luciferase reporter assay to determine if CD47 is directly targeted by miR-96-5p. Using TargetScan, two possible binding sites were identified (Fig. 2E), the first of which was highly conserved in primates (Fig. 2 F). We subcloned various 3′-UTR segments, each featuring either a wild-type or mutant binding site, into the pmirGLO vector (Fig. 2G). According to Fig. 2H, miR-96-5p reduced luciferase activity by about 40% at site 1 and 20% at site 2; anti-miR-96-5p marginally increased activity at site 1; mutating both sites to disrupt potential base pairing nullified miR-96-5p’s impact on luciferase activity. The data demonstrated that miR-96-5p directly targets and negatively regulates CD47 expression.

### miR-96-5p-mediated downregulation of cell surface CD47 facilitates phagocytosis of PDAC cells by macrophages

CD47-SIRPα signaling is crucial for phagocytosis by human and murine macrophages in many hematologic and solid malignancies. Since CD47 is miR-96-5p’s direct target, we investigated how it affects macrophages’ ability to engulf cancer cells. Firstly, we examined miR-96-5p’s capacity to facilitate the phagocytosis of PDAC cells by human macrophages in vitro. Phagocytosis was assessed by coculturing eFluor™ 670-labeled THP1-derived macrophages with CFSE-labeled PDAC cells, followed by FACS analysis. Our study observed an increase in phagocytosis of PDAC cells with miR-96-5p mimics, whereas a decrease was noted with the use of inhibitors (Fig. 2I).

To further assess the impact of miR-96-5p on phagocytosis, we employed CRISPR/Cas9 technology to create miR-96-5p^−/−^ PDAC cells (Fig. 2J). PCR identification and DNA sequencing confirmed ablation of endogenous miR-96-5p (Fig. 2J and Fig. S3). MiR-96-5p^−/−^ cells exhibited elevated CD47 protein levels compared to wild-type cells (Fig. 2K). Macrophages were cocultured with wild-type and miR-96-5p^−/−^ cells, with or without mimics, for in vitro phagocytosis experiments. As expected, miR-96-5p deletion lowered phagocytosis efficiency, but retrieving miR-96-5p rescued it (Fig. 2L). I*n vitro* phagocytosis assays were also performed with mouse macrophages. Mouse macrophages derived from the bone marrow of C57BL/6 mice’s femur and tibia were induced with 10 ng/mL M-CSF over 5 days and subsequently cocultured with Panc02. Similar to human macrophages, BM-derived macrophages phagocytized Panc02 cells more when miR-96-5p overexpression. Panc02 cells’ phagocytosis was greatly reduced by miR-96-5p suppression or deletion (Fig. S4). The results indicate that miR-96-5p is crucial for CD47-mediated macrophage phagocytosis.

### Restoration of miR-96-5p in PDAC cells exerts anti-tumor activity via promoting M1 polarization of macrophages and CD8^+^T cell responses in a subcutaneous model

**Fig. 3:**
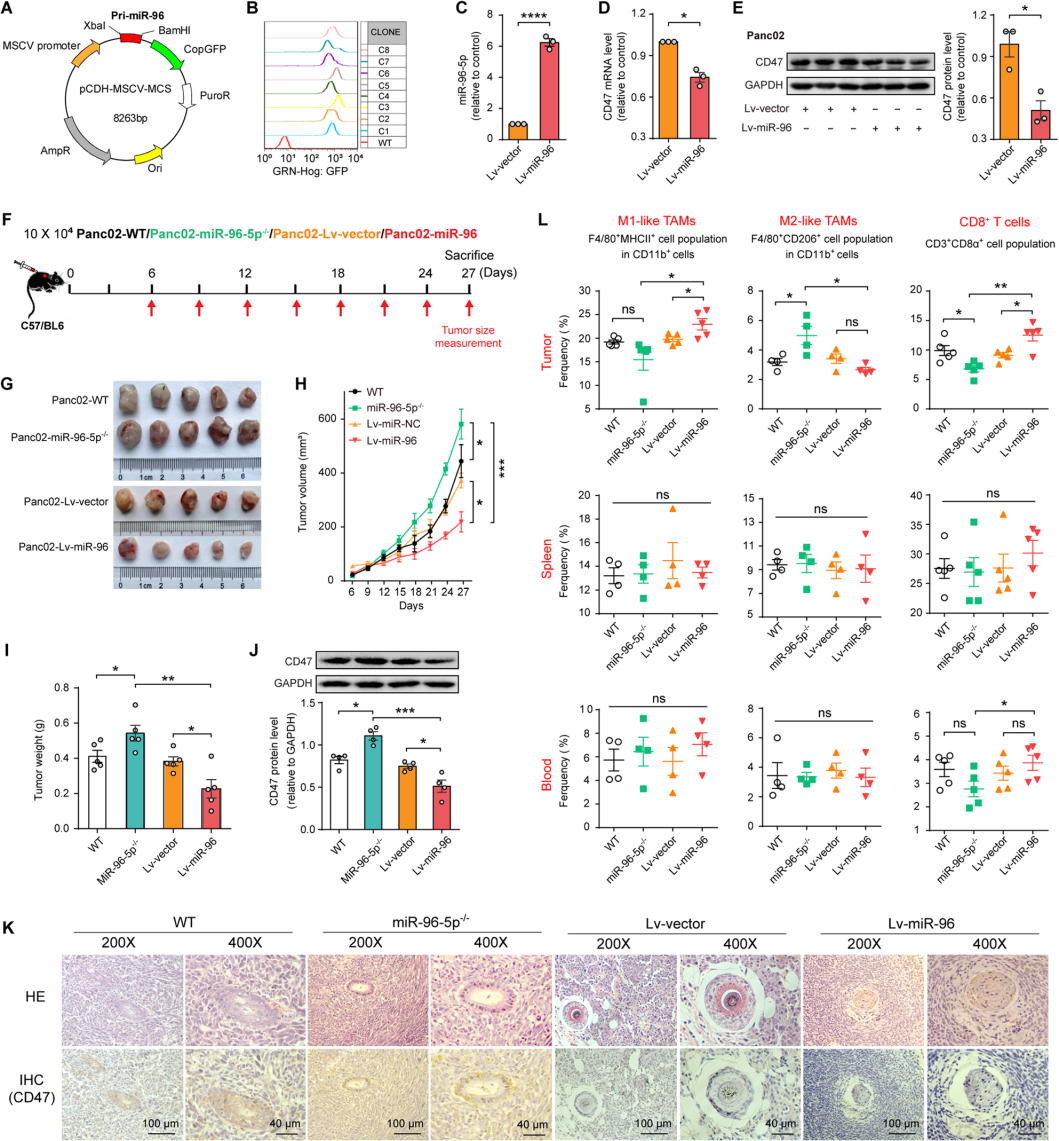
Restoration of miR-96-5p in PDAC cells exerts anti-tumor activity via promoting M1 polarization of macrophages and CD8^+^ T cell responses in a subcutaneous model. **A** The Pri-mmu-miR-96 segments were amplified and inserted into the *XbaI* and *BamHI* cleavage sites of the lentiviral vector. **B** The high-expression monoclonal was selected by detecting GFP using FACS. **C** qPCR analysis of miR-96-5p levels in Panc02-Lv-vector or -Lv-miR-96 (*n* = 3). **D-E** qPCR and Western blot examinations of CD47 expression in Panc02-Lv-vector or -Lv-miR-96 cells (*n* = 3). **F** Schematic illustration of the experiment schedule. **G** Images of stripped tumors from mice bearing Panc02 cells with wild-type, miR-96-5p^−/−^, Lv-vector, and Lv-miR-96. **H** Mouse pancreatic tumor growth rate (*n* = 5). **I **Tumor weight (*n* = 5). **J** Analysis of CD47 levels in cancerous tissues (*n* = 4). **K** Representative IHC images of CD47 expression in tumor samples. **L** Quantitation of M1/M2-like macrophages and CD8^+^ T cells was performed in tumors, spleen, and blood of mice implanted with Panc02 cells, including wild-type, miR-96-5p^−/−^, Lv-vector, and Lv-miR-96 variants (*n* = 4)

To delve deeper into the impact of miR-96-5p enforced expression on the development of PDAC, we performed a subcutaneous PDAC model experiment in immunocompetent C57BL/6 mice. Stably transfecting cells with a lentivirus containing mmu-pri-miR-96 created a high-expressing cell line, Lv-miR-96 (Fig. 3 A), and the high-expression monoclonal was selected by FACS and confirmed by qPCR (Fig. 3B and C). In Panc02 cells, ectopic miR-96-5p expression obviously lowered CD47 mRNA and protein levels (Fig. 3D and E). In the xenograft mouse model, once tumors were noticeable, vernier calipers measured their diameters every three days (Fig. 3 F). Figure 3G showed that miR-96-5p upregulation notably suppressed tumor progression, evidenced by a marked decrease in tumor size (Fig. 3G), growth speed (Fig. 3H), and weight (Fig. 3I) compared to the other control groups, especially the group with miR-96-5p deletion. Western blot analysis revealed that CD47 levels were elevated in the miR-96-5p deletion group compared to the wild-type and lentiviral empty vector control groups, whereas overexpression of miR-96-5p resulted in reduced CD47 levels (Fig. 3 J). CD47 staining in paraffin sections was identical (Fig. 3 K).

Many reports have shown that CD47 overexpression could provoke an immunosuppressive microenvironment by polarizing macrophages to M2 [35, 36]. FACS assays were performed on mice’s tumors, spleens, and peripheral blood to evaluate the accumulation of immune cell subsets (Fig. S5), specifically M1/M2 macrophages (CD11b^+^F4/80^+^MHC-Ⅱ^+^, CD11b^+^F4/80^+^CD206^+^), CD4^+^T (CD3^+^CD4^+^), CD8^+^T (CD3^+^CD8^+^), and NK (CD3^+^NK1.1^+^) cells. We observed that miR-96-5p mainly affected the distribution of immune cells in tumor tissues, while that in the spleen and peripheral blood was similar to that in the control group. In the tumor tissues, we found that the activated state of TAMs in the tumors of mice bearing Lv-miR-96 Panc02 cells was predominantly M1-type, and the abundance of CD8^+^ cytotoxic T cells (CTLs) was also higher, compared with the tumors of mice bearing Lv-vector and miR-96-5p deficient cells. In peripheral blood, the Lv-miR-96 group exhibited a marginally higher proportion of CD8^+^ T cells compared to the miR-96-5p^−/−^ group (Fig. 3L). No significant statistical differences were observed in CD4^+^ T and NK cell populations across the tumor, spleen, and peripheral blood among the four groups (Fig. S6). Our in vivo findings reinforce the tumor-suppressive function of miR-96-5p in PDAC by promoting antitumor immunity, as evidenced by M1 macrophage polarization and activation of antitumor effector CD8^+^ T cells within the tumor microenvironment.

### miR-96-5p associates with immune response and cell growth, and it mediates M1 polarization of macrophages via exosomal transfer

**Fig. 4:**
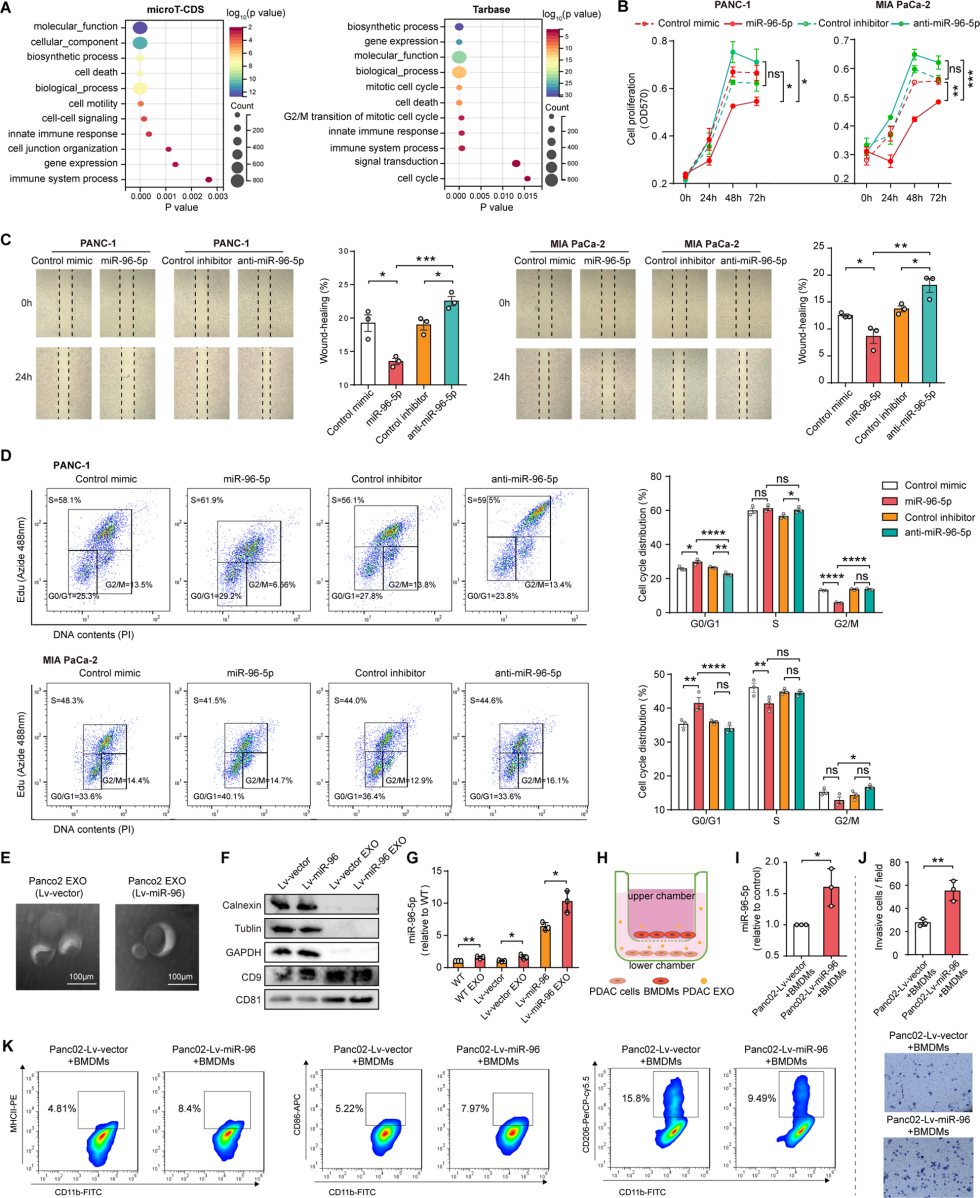
miR-96-5p is linked to immune response and cell growth, and it mediates M1 polarization of macrophages via exosomal transfer. **A** GO enrichment analysis of miR-96-5p in terms of biological processes using DIANA tools on the basis of public microT-CDS and Tarbase datasets. **B** MTT assays assess the proliferation of PDAC cells post-transfection with miR-96-5p or anti-miR-96-5p (*n* = 3). **C** Wound healing assays assess the migration of PDAC cells with miR-96-5p overexpression or inhibition. Images of the cells were taken at 0 and 24 h, and the gap width was calculated using ImageJ (*n* = 3). **D** EdU/PI cell cycle analysis. PDAC cells with miR-96-5p overexpression or inhibition and pulse-labeled with EdU for 2 h before Alexa 488 fluorescent labeling, PI staining, and FACS analysis (*n* = 3). **E** Transmission electron microscopy image depicting exosomes derived from PDAC cells. **F** Western blot examination showed calnexin, Tubulin, GAPDH CD9, and CD81 in Panc02 cells (Lv-vector and Lv-miR-96) and Panc02 cell-derived exosomes (Lv-vector EXO and Lv-miR-96 EXO) (*n* = 3). **G** MiR-96-5p expression in both PDAC cells and their corresponding exosomes (*n* = 3). **H** Illustration of BM-derived macrophages co-cultured with PDAC cells in a transwell co-culture system. **I** Assessment of miR-96-5p levels in the co-culture medium (*n* = 3). **J-K** Effects of exosomes derived from Panc02 (Lv-vector and Lv-miR-96) on macrophage recruitment (**J**) and polarization (**K**) (*n* = 3)

To clarify the mechanism of miR-96-5p responsible for anti-tumor immune, we conducted GO analysis with DIANA-miRPath v3.0, leveraging TarBase and microT-CDS datasets to pinpoint biological process pathways potentially affected by miR-96-5p. The results suggest a strong correlation between miR-96-5p and both cell growth and immune response (Fig. 4 A). Thereafter, we study PDAC, focusing on exploring these biological functions. MTT assays verified that miR-96-5p significantly suppressed the proliferation of PDAC cells, whereas anti-miR-96-5p showed no noticeable effect (Fig. 4B). Wound healing assay in Fig. 4 C showed that, migration was suppressed by 75 nM miR-96-5p transfection, but endogenous inhibition facilitated it. Cell cycle analysis (Fig. 4D) indicated that miR-96-5p transfection led to an increase in G0/G1 phase cells and a decrease in G2/M phase cells compared to the control transfection. Compared to control-transfected cells, anti-miR-96-5p-transfected PDAC cells showed fewer G0/G1 cells and more G2/M cells.

Given that exosomes enable communication between tumor and immune cells, we examined whether exosomes produced from PDAC cells induce M1 polarization of macrophages. Isolated particles from Panc02-Lv-vector and Panc02-Lv-miR-96 cell culture supernatants were identified as exosomes by electron microscopy (Fig. 4E). Western blotting revealed that the exosome markers CD81 and CD9 were strongly expressed, whereas calnexin, Tubulin and GAPDH were not (Fig. 4 F). Furthermore, exosomes showed a notable elevation of miR-96-5p, highlighting their capacity for miRNA enrichment, as evidenced by the analysis of miR-96-5p levels in Panc02, Panc02-Lv-vector, Panc02-Lv-miR-96 cells, and their exosomes (Fig. 4G). M0 macrophages cocultured with Panc02 cells in transwell chambers exhibited elevated miR-96-5p levels (Fig. 4H-I). Interestingly, there was more BMDM migration in the Lv-miR-96 group, suggesting that miR-96-5p may promote macrophage recruitment in the form of exosomes (Fig. 4 J). In addition, macrophages in the Lv-miR-96 group showed more M1 marker expression and less M2 marker expression (Fig. 4 K). Taken together, the findings suggest that restoration of miR-96-5p in PDAC cells inhibits tumor cell growth and promotes macrophage recruitment and M1 polarization via exosomal transfer.

### Macrophages enhance antigen cross-presentation after phagocytosis of PDAC cells with miR-96-5p overexpression

**Fig. 5:**
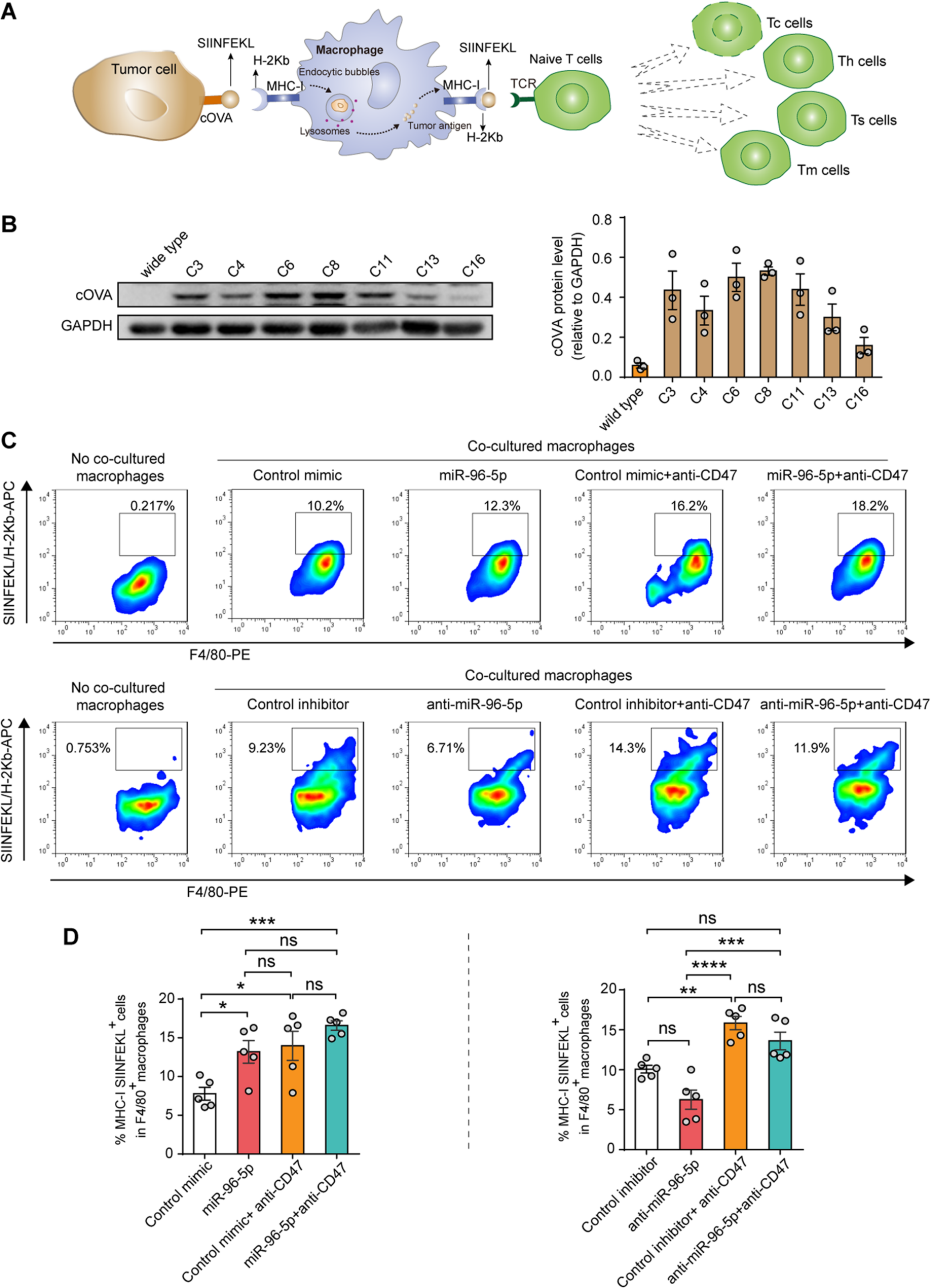
Following the phagocytosis of PDAC cells via miR-96-5p overexpression, BM-derived macrophages enhance antigen cross-presentation. **A** Diagram of macrophage-mediated antigen presentation and T cell activation. **B** Western blot examination of the OVA protein levels (*n* = 3). **C-D** After Panc02-cOVA cells were treated with or without anti-CD47 mAb, the effect of miR-96-5p on antigen presentation was assessed using Guava EasyCyte™ FACS (*n* = 5)

Macrophages, functioning as APCs, can process ingested tumor cells and present their antigens to T cells, thus triggering antigen-specific immune responses [15, 37] (Fig. 5A). In vivo experiments revealed CD8^+^ T cell activation in tumor tissues with elevated miR-96-5p expression, indicating that miR-96-5p may enhance macrophage-mediated antigen cross-presentation through increased tumor cell clearance. We evaluated the hypothesis by coculturing Panc02-cOVA cells (Clone 8, Fig. 5B), transfected with miR-96-5p mimics or inhibitors and treated with or without 10 µg/mL anti-CD47 mAb, alongside BMDMs to assess the presence of MHC-I-bound SIINFEKL. As anticipated, a notable increase in the proportion of SIINFEKL from BMDMs was observed in the miR-96-5p transfection group, with an even greater increase in the group co-treated with anti-CD47 mAb. However, anti-miR-96-5p partially inhibited the antigen cross-presentation effect (Fig. 5C and D). Together, these results suggest that increased phagocytosis through re-introduction of miR-96-5p expression also promoted antigen cross-presentation by macrophages.

### Macrophages prime OT-I/OT-II (CD8^+^/CD4^+^) T cells after phagocytizing PDAC cells with miR-96-5p overexpression

**Fig. 6:**
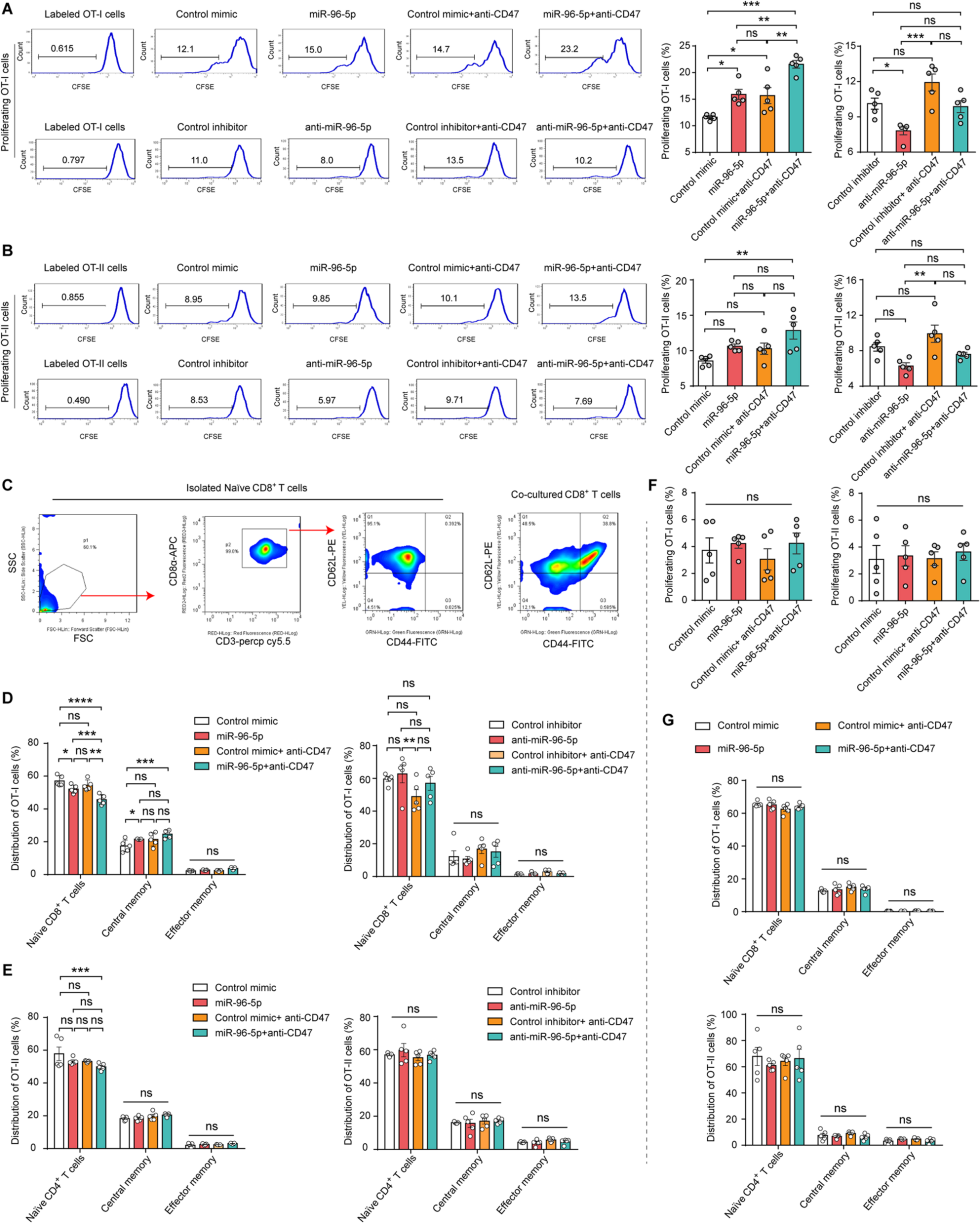
Macrophage phagocytosis-induced antigen cross-presentation promotes T-cell priming. **A-B** FACS examinations of effect of miR-96-5p on the proliferation of CD8^+^ (**A**) and CD4^+^ (**B**) T cells cocultured with BMDMs and Panc02-cOVA cells, with and without anti-CD47 mAb treatment (*n* = 5). **C** Gating strategies for identifying T cell maturation. **D-E** FACS analyses of effect of miR-96-5p on the maturation of CD8^+^ (**D**) and CD4^+^ (**E**) T cells (*n* = 5). **F-G** Without macrophages, tumor cells and T cells were co-cultured for 72 h to evaluate the impact of miR-96-5p mimics and anti-CD47 mAb on CD8^+^/CD4^+^ T cell proliferation (**F**) and maturation (**G**) (*n* = 5)

Considering the crucial role of antigen cross-presentation by professional APCs in T-cell priming, we next investigated whether miR-96-5p boosts the effective priming of T cells specific to antigens. We accomplished this by introducing naïve CD8^+^ or CD4^+^ T cells (labeled with CFSE) into cocultures of BM-derived macrophages and Panc02-cOVA cells (treated with miR-96-5p mimics/inhibitors or anti-CD47 mAb) for 3 days. FACS was used to analyze CFSE dye dilution in CD4^+^T and CD8^+^T cell populations. In our system, miR-96-5p and anti-CD47 mAb have significant promoting effects on CD8^+^ T-cell activation, and their combined effect is more powerful, while anti-miR-96-5p inhibits CD8^+^ T cell activation (Fig. 6A). Neither miR-96-5p mimics/inhibitors, nor anti-CD47 mAb alone significantly affected CD4^+^ T cell proliferation; however, the combination of miR-96-5p mimics and anti-CD47 mAb substantially enhanced CD4^+^ T cell proliferation (Fig. 6B).

Subsequently, FACS was employed to evaluate T cell maturation, identifying naïve T cells as CD44^low^CD62L^high^, central memory T cells as CD44^high^CD62L^high^, and effector memory T cells as CD44^high^CD62L^low^. After the naïve T cells were extracted from the spleens of OT-I and OT-II mice, we first detected their purity and maturity and found that almost 100% of the extracted cells were target cells and most of them were CD44^low^ CD62L^high^ naïve T cells. Moreover, co-culturing the extracted T cells with Panc02-cOVA cells and macrophages resulted in their differentiation (Fig. 6C). We found that after transfection with miR-96-5p, a fraction of OT-I T cells matured from a naïve phenotype (CD44^high^CD62L^low^) toward a central memory (CD44^high^ CD62L^high^), and exerted a stronger promoting role when joint treatment with anti-CD47 mAb. However, anti-CD47 mAb alone did not directly promote T cell maturation. CD8^+^ T cell maturation was unaffected by anti-miR-96-5p transfection (Fig. 6D). For CD4^+^ T cell maturation, we only observed that the combination of miR-96-5p and anti-CD47-mAb significantly reduced the ratio of naïve CD4^+^ T cells (Fig. 6E). We posed the question of whether T cell priming was independent of the enhanced antigen presentation resulting from the augmented phagocytosis of macrophages mediated by miR-96-5p overexpression, or if the overexpression of miR-96-5p in tumor cells could directly activate T cells. To this end, we only co-cultured the extracted naïve T cells with Panc02-cOVA cells (transfection with miR-96-5p). The findings indicated that neither miR-96-5p overexpression, anti-CD47 mAb treatment, nor their combination directly influenced T cell proliferation (Fig. 6F) or maturation (Fig. 6G). Overall, these results revealed that miR-96-5p-mediated phagocytosis of PDAC cells predominantly primes CD8^+^ T cells, and the linkage between innate (tumor cell phagocytosis due to CD47 inhibition by miR-96-5p overexpression) and adaptive immune responses (T-cell priming) is mediated by phagocytosis-induced antigen cross-presentation.

### Administration of AAV carrying miR-96-5p activates more of the immune response in a metastatic model

**Fig. 7:**
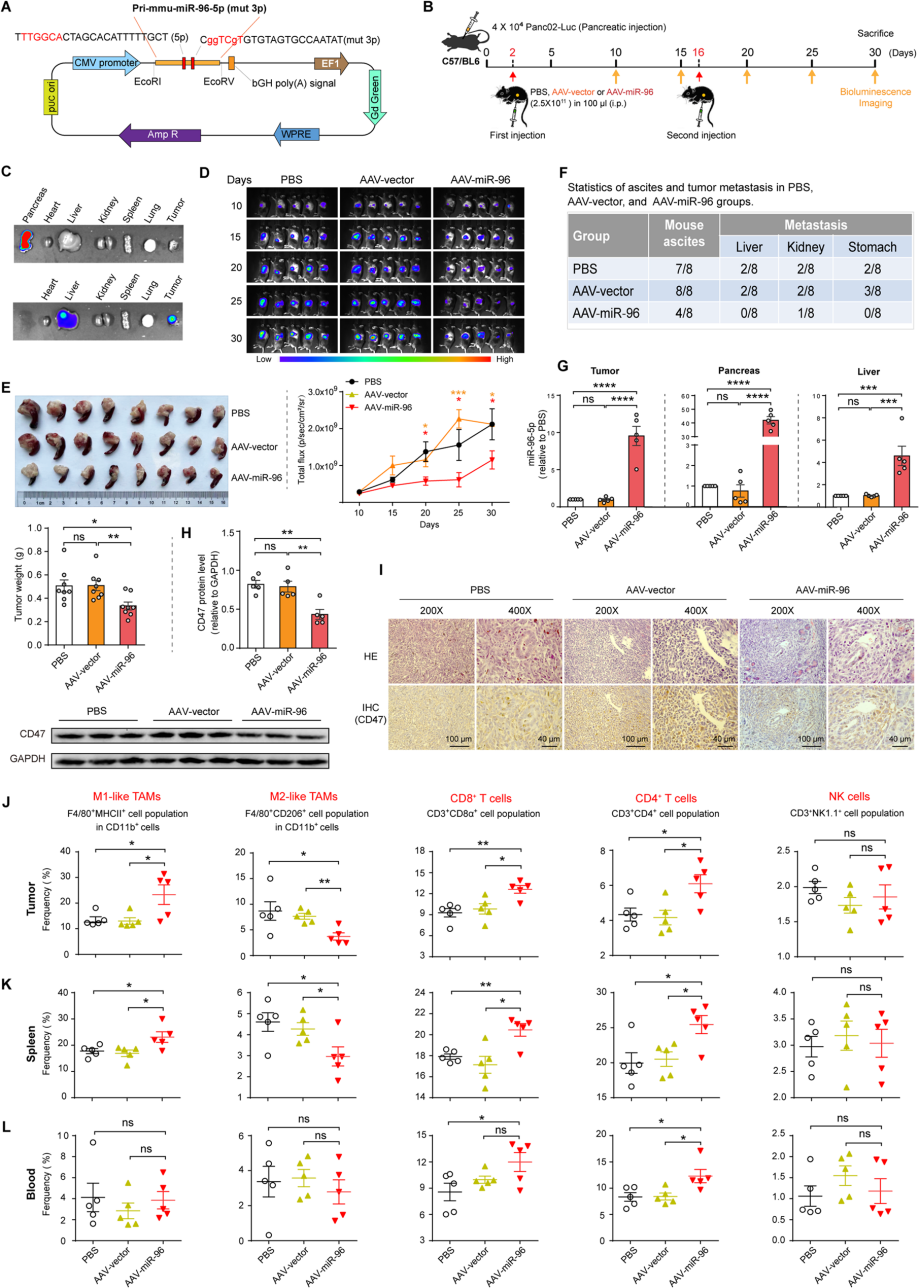
AAV-mediated expression of miR-96-5p activates more of the immune response in a metastatic model. **A** Illustration of AAV vectors designed to express miR-96-5p exclusively, excluding miR-96-3p. 500 bp of pri-mmu-miR-96 was inserted downstream of the CMV promoter. **B** Scheme for AAV administration in tumor-bearing murine models. Mice bearing orthotopic PDAC created with Panc02-luc were subjected to i.p. injection with PBS, AAV-vector, or AAV-miR-96 on days 2 and 16 of the Panc02 injection and IVIS imaging every five days on day 10 of the tumor cell injection. **C** IVIS system detection of GFP expression in mouse tissues and organs. Top: including the pancreas. Bottom: excluding the pancreas. **D** Bioluminescent images of mice every five days by IVIS imaging and quantitative analysis of the results of bioluminescence imaging (*n* = 5). **E** Tumor images and tumor weights of the indicated groups at the end of experiments (*n* = 8). **F** Statistical analysis of ascites and pancreatic tumor metastasis in the liver, kidney and stomach of mice (*n* = 8). **G** qPCR analysis of miR-96-5p levels in pancreas, pancreatic tumor and liver tissues (*n* = 5). **H** Western blot analysis of CD47 expression in tumors. **I** IHC images illustrating CD47 expression in tumor samples (*n* = 5). **J-L** The frequencies of M1/M2-like TAMs, CD8^+^ T, CD4^+^ T, and NK cells were analyzed in the tumor microenvironment (**J**), spleen (**K**), and blood (**L**) of mice from the specified groups (*n* = 5)

To translate our findings to a therapeutic model, we also explored the ability of miR-96-5p to eliminate PDAC in vivo through drug administration strategies. An orthotopic metastatic pancreatic cancer model was developed by inoculating Panc02-luc cells into the pancreas of C57/BL6 mice. To deliver miR-96-5p into tumors in the mouse pancreas as targeted as possible, we generated a recombinant AAV-PAN-CMV-GFP vector expressing the primary sequence of the murine miR-96-5p (pri-mmu-miR-96-5p, mut 3p) driven by the constitutive cytomegalovirus (CMV) promoter (Fig. 7A). Specifically, we administered i.p. injections on days 2 and 16 following the orthotopic transplantation of tumor cells. On the 10th day, mice were imaged with the IVIS system every five days (Fig. 7B). To investigate the morphological effects of AAV-PAN on tissues and organs, we collected major tissues and organs from mice for HE staining. The study found no significant morphological differences in the pancreas, heart, liver, spleen, lung, and kidney between mice injected with AAV-vector and AAV-miR-96 viruses and those injected with PBS (Fig. S7A). We also used the IVIS system and fluorescence microscope to identify the targeting of AAV-PAN to various tissues and organs in mice. Results demonstrated robust GFP expression in the pancreas of mice injected with the virus, as well as increased GFP expression in the pancreatic tumor and liver (pancreas > pancreatic tumor > liver) (Fig. 7C and Fig. S7B). Fig. 7D-F illustrated that miR-96-5p overexpression substantially ameliorated PDAC progression. This was evidenced by a dramatic reduction in intensity of bioluminescent signals (Fig. 7D), tumor size and weight (Fig. 7E), as well as the occurrence of ascites and tumor metastasis (Fig. 7F and Fig. S8). qPCR analysis of miR-96-5p levels showed that compared with the control group, the levels of pancreatic tumors, pancreas and liver in mice of the AAV-miR-96 group were approximately 10 times, 50 times and 5 times higher, respectively (Fig. 7G). Immunohistochemistry and Western blotting followed, both of which showed significantly reduced CD47 protein abundance in the miR-96-5p overexpression group (Fig. 7H and I). Moreover, FACS analysis of immunocyte subsets revealed that the administration of AAV carrying miR-96-5p resulted in a heightened immune response (Fig. 7J-L). Specifically, the activation state of TAMs in both the tumor and spleen of mice in the AAV-miR-96 group was predominantly M1 type, with a relatively minimal presence of M2 type. Furthermore, in the AAV-miR-96, there was a notable rise in the proportion of CD8^+^ T cells and CD4^+^ T cells within CD3^+^ lymphocytes in the tumor, spleen, and peripheral blood. However, no significant differences in NK cells were observed. The findings indicate that the AAV-miR96 virus can successfully suppress the growth of pancreatic tumors in situ and activate more anti-tumor immunity.

## Discussion

The high mortality and poor prognosis of PDAC primarily result from challenges in early diagnosis and ineffectiveness of treatments for advanced stages. Understanding the pathogenesis and molecular changes in PDAC is crucial for developing new diagnostic biomarkers, effective treatments, and improving prognosis. The active CD47/SIRPα signaling pathway drives evasion of immune surveillance in a variety of malignancies and therefore poses a greater challenge for cancer treatment, including PDAC [38, 39]. Emerging clinical trials are examining the effectiveness of anti-CD47 mAbs and SIRPα-Fc fusion proteins in various cancers [13, 15]. Current options encompass CD47 mAbs like Hu5F9-G4 (Gilead Sciences, Inc.), TJC4 (I-Mab Biopharma, Co., Ltd.), AO-176 (Arch Oncology, Inc.), and IBI188 (Innovent Biologics, Inc.), along with SIRPα-Fc fusion proteins such as ALX148 (ALX Oncology, Inc.) and TTI-621 (Trillium Therapeutics, Inc.). However, these treatments often result in various adverse events (anemia, thrombocytopenia, nausea, diarrhea, et al.), and their effectiveness may be compromised due to limited tissue infiltration capacity and inadequate immune activation [40, 41]. Nowadays, miRNA-based therapeutics have become essential, particularly for immunological regulation [42].

By targeting CD47, miR-708 has been identified as a promoter of phagocytosis in T-cell acute lymphoblastic leukemia [43], and miR-340 restoration boosts macrophage phagocytosis and improves T cell effector functions in PDAC [44]. Herein, a new suppressor of CD47, miR-96-5p, was discovered. Human miR-96, located on chromosome 7 at locus 7q32.2, and murine miR-96, found on chromosome 6 at locus 6 A3.3, both mature into − 5p and − 3p forms [45]. Although aberrant expression of miR-96-5p has been linked to the initiation and development of multiple malignancies [46, 47], its mechanism in PDAC remains largely elusive, especially in immunity. Cox regression analysis of TCGA and CPTAC PDAC data indicated a strong correlation between low miR-96-5p levels in PDAC tissues and poor clinical prognosis. CD47 is overexpressed in PDAC tissues, implying that reduced miR-96-5p levels may lead to CD47 upregulation and subsequent antitumor immunosuppression. Like miR-340 and miR-708, miR-96-5p remodeled the immune surveillance function of macrophages. Restoration of miR-96-5p in PDAC cells substantially enhanced degradation of CD47 mRNA and protein, thereby promoting macrophage-mediated phagocytic clearance of PDAC cells. TAMs, as the predominant immune cells within tumor environments, are crucial in modulating antitumor immunity [48]. Lu et al. reported that upregulation of CD47 could facilitate tumor immune escape by influencing the polarization of macrophages [35]. TAM can differentiate into two contrasting phenotypes: classically activated M1-like macrophages display pro-inflammatory and tumoricidal qualities, whereas M2-like macrophages are alternatively polarized and possess anti-inflammatory and tumor-promoting attributes [49]. These phenotypes markedly influence tumor progression and clinical outcomes [50]. A prior study even demonstrated that anti-CD47 treatment alone can induce macrophages to adopt an M1-like phenotype in vivo, enhancing their antitumor activity [51], suggesting a relationship between the CD47-SIRPα pathway and reprogramming of macrophage polarization. Our results are consistent with previous reports showing that the activation state of TAMs in miR-96-overexpressing tumors was predominantly M1-type, whereas that in the miR-96-5p-deleting tumors was mainly M2-type. Exosomes originating from tumor cells are key regulators of the complex interplay between tumor cells and tumor microenvironment (TME) cells, influencing tumor progression by modulating the phenotype and function of TME cells [52, 53]. Herein, mechanism exploration suggested that elevated miR-96-5p expression in PDAC can promote macrophage recruitment and M1 polarization through exosomal transfer of miR-96-5p. Additionally, our research revealed that tumors with stable overexpression of miR-96-5p exhibited a high frequency of CD8^+^ cytotoxic T cells among CD3^+^ lymphocytes, whereas tumors with miR-96-5p deficiency were significantly decreased. Macrophages, essential APCs, play a crucial role in the innate immune system and serve as a bridge to the adaptive immune system. We hypothesize that miR-96-5p enhances macrophage phagocytosis, potentially presenting tumor antigens to T cells and initiating T cell priming. In the ovalbumin model antigen system, assays for antigen presentation and T cell proliferation/maturation demonstrated that miR-96-5p-enhanced removal of cancer cells led to increased antigen presentation and priming of T cells, particularly CD8^+^ T cells. Notably, the group combining CD47mAb and miR-96-5p demonstrated much greater efficiency. With the assistance of miR-96-5p, the combined treatment not only enhanced macrophage phagocytic and T cell activation capabilities but also directly inhibited tumor cell proliferation and invasion. This multi-pathway synergistic effect, through enhanced efficacy and complementary mechanisms, demonstrates higher practical value and provides a new strategy for PDAC treatment. More recent studies also showed that anti-CD47 therapies in mouse tumor models activate adaptive immune responses [14, 15, 38]. A study by Feng et al. outlined that CD47 blockade could enhance tumor cell phagocytosis by DCs, thereby activating innate immunity and subsequently triggering an adaptive immune response that leads to tumor regression [54]. Consequently, DCs might significantly contribute to miR-96-5p-mediated anti-tumor immunity, warranting further investigation in extensive future studies.

With the emerging evidence that miR-based therapies have enormous potential for treating various diseases, there has been a drastic surge of interest in them over the last few decades. Therapeutic approaches are advancing, such as chemically modifying two-stranded miRNA mimics or integrating them with polyethyleneimine (PEI) [55], liposomes [56], nanoparticles [57], or virus particles [58]. However, delivering miRNA safely and effectively to target cells or tissues remains a major challenge due to its low tissue permeability, non-targeting, susceptibility to degradation by nucleases, and fast removal from the blood [59]. To overcome these difficulties as much as possible, our studies initially assessed the anti-tumor effectiveness of miR-96-5p in genetic approaches (knockout and overexpression), subsequently examined its effectiveness using a drug delivery system based on AAV-PAN viruses [34], and provided evidence that the AAV-PAN system can partially target pancreatic tumors. Besides, the high expression of miR-96-5p mediated by AAV-PAN had no pathological effect on the tissues and organs of mice. Most notably, in the orthotopic tumor model, there was a greater activation of anti-tumor immune responses compared to the subcutaneous model. Nonetheless, enhancing the specificity and efficacy of the AAV-PAN virus for penetrating pancreatic tumor cells, maintaining AAV transgenic activity in rapidly proliferating tumor cells, and achieving broader tumor cell coverage requires further refinement. Another major problem is the off-target effects caused by miRNAs’ multi-targeting feature, which occurs when miRNA interacts with mRNA that is not its intended target, leading to unexpected biological outcomes. A first-in-human phase 1 trial evaluating MRX34 [56], a miR-34a mimic encapsulated in liposome nanoparticles, focused on patients with advanced solid tumors, such as melanoma, renal cancer, and non-small cell lung cancer. Despite the notable efficacy of MRX34, the clinical trial was discontinued due to severe immune-mediated adverse events (NCT01829971), partially attributable to off-target effects. To reduce off-target effects, in the future, miR-96-5p analogues modified with 2’-O-methyl (2’-O-Me) [60] or locked nucleic acid (LNA) [61] may be used to enhance their base pairing affinity and specificity, thereby balancing their efficacy and safety.

**Fig. 8:**
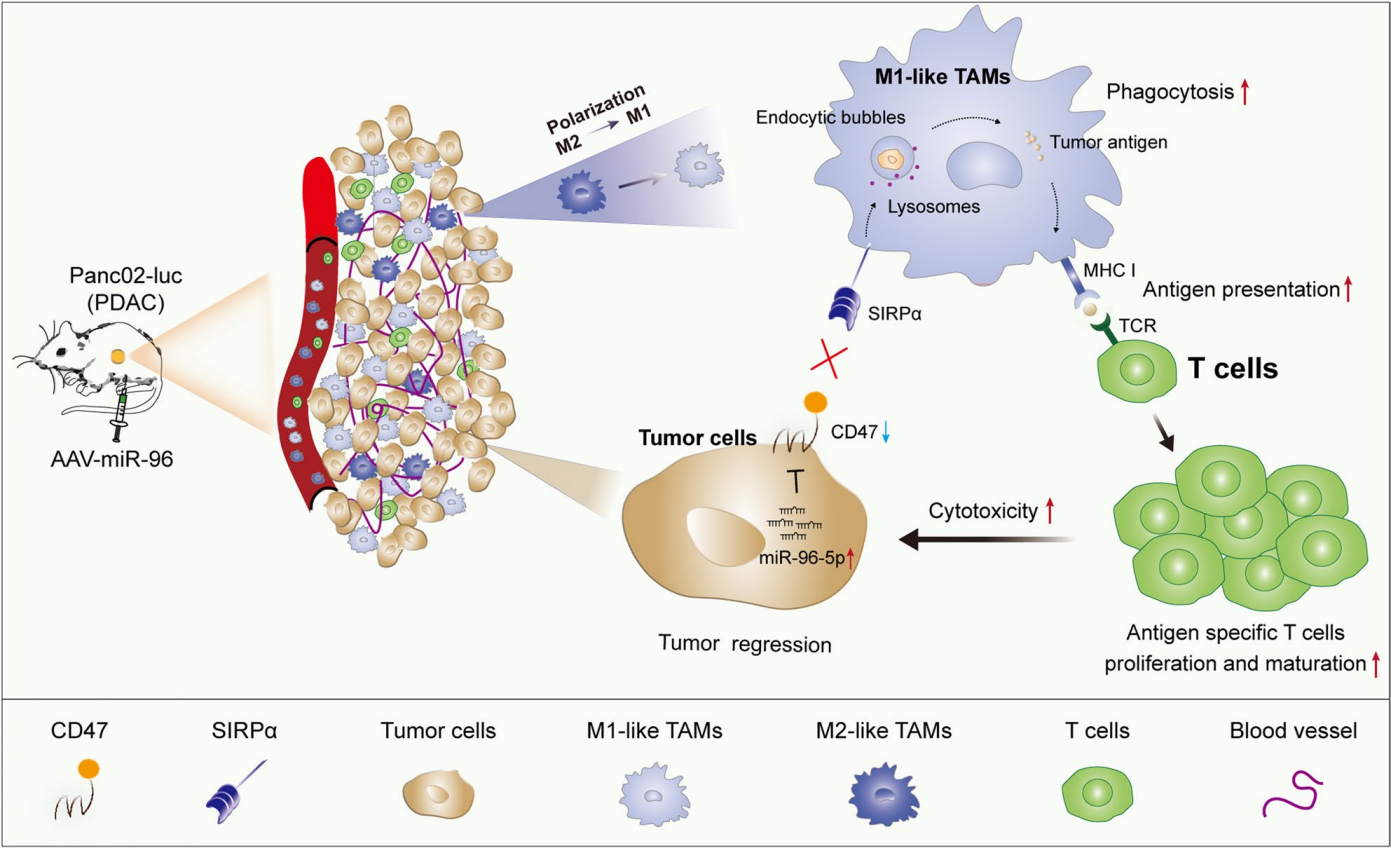
The schematic diagram depicts the molecular mechanism by which miR-96-5p inhibits tumor growth through the activation of anti-tumor immunity

In conclusion, restoration of miR-96-5p in pancreatic tumors can promote phagocytic clearance of tumor cells by inhibiting CD47 (innate immune activation), thereby enabling macrophages to present more tumor antigens to T cells and supporting T cell proliferation and maturation (adaptive immune activation), ultimately resulting in tumor regression (Fig. 8). Activation of the innate and adaptive immune responses against cancer may enable miR-96-5p to be a prospective therapeutic target for PDAC.

## Supplementary Information

Supplementary material 1.

Supplementary material 2.

## Data Availability

The data that support the findings of this study are available on request from the corresponding author.
