# Supplementary material for: miR-96-5p-mediated Inhibition of CD47 contributes to pancreatic tumor regression via activating both innate and adaptive anti-tumor immunity
Source: Cell Commun Signal. 2025 Dec 5;24:16. doi: 10.1186/s12964-025-02582-5 (PMC12797448; doi:10.1186/s12964-025-02582-5)
Supplement: Supplementary file 1 — Supplementary material 1. [file 12964_2025_2582_MOESM1_ESM.docx]

**Supplementary Materials**

**Supplementary Tables**

**Table S1.** miRNA mimic and inhibitor sequences for cell transfection.

| miRNA mimics and inhibitors |  | miRNA mimic and inhibitor sequence (5′-3′) |
| --- | --- | --- |
| Control mimics sense |  | UUCUCCGAACGUGUCACGUTT |
| Control mimics antisense |  | ACGUGACACGUUCGGAGAATT |
| miR-96-5p mimics sense |  | UUUGGCACUAGCACAUUUUUGCU |
| miR-96-5p mimics antisense |  | CAAAAAUGUGCUAGUGCCAAAUU |
| Control inhibitors |  | CAGUACUUUUGUGUAGUACAA |
| miR-96-5p inhibitors |  | AGCAAAAAUGUGCUAGUGCCAAA |

**Table S2.** Sequence of primers used for qPCR.

| Gene | Primer sequence (5′-3′) | | |  | Product size (bp) |
| --- | --- | --- | --- | --- | --- |
|  | Forward |  | Reverse |  |  |
| human-CD47 | GGCAATGACGAAGGAGGTTA |  | ATCCGGTGGTATGGATGAGA |  | 217 |
| human-GAPDH | GGAAGGTGAAGGTCGGAGTC |  | TGGAATTTGCCATGGGTGGA |  | 166 |
| mouse-CD47 | GCTTCTGGACTTGGCCTCAT |  | CCTCTGGTTGGAAGCGACAA |  | 273 |
| mouse-GAPDH | AGGTCGGTGTGAACGGATTTG |  | TGTAGACCATGTAGTTGAGGTCA |  | 129 |
| miR-96-5p | TATGTGGGATGGTAAACCGCTT |  | Universal PCR Primer R |  | -- |
| U6 | U6 Primer F |  | Universal PCR Primer R |  | -- |

**Table S3.** Amplified primer sequences of mouse pri-miR-96 fragment and CD47 3'-UTR fragment. (F, forward primer; R, reverse primer)

| Primer Name |  | Primer sequence (5′-3′) |  | Restriction enzyme |
| --- | --- | --- | --- | --- |
| Pri-miR-96 F |  | CTAGTCTAGAGGCACTGGTAGAATTCACT |  | *XbaI* |
| Pri-miR-96 R |  | CGCGGATCCAACAGGGCATCACAGAAG |  | *BamHI* |
| Position 1599-1605 of CD47 3′-UTR F |  | CGAGCTCGGTGGGGTACCTACAGGAG |  | *SacI* |
| Position 1599-1605 of CD47 3′-UTR R |  | CCGCTCGAGAAAGTGCTGCAGGAGCCATC |  | *XhoI* |
| Position 1599-1605 of CD47 3′-UTR mutant F |  | GAAATATGACAATCAATATCGACTAAAAGAGGCACAC |  | *--* |
| Position 1599-1605 of CD47 3′-UTR mutant R |  | GTGTGCCTCTTTTAGTCGACATTGATTGTCATATTTC |  | *--* |
| Position 2504-2510 of CD47 3′-UTR F |  | CGAGCTCTGGCTCCCAAATTCCATCACA |  | *SacI* |
| Position 2504-2510 of CD47 3′-UTR R |  | CCGCTCGAGAAGGAGAAGAGCAAGGCCAAA |  | *XhoI* |
| Position 2504-2510 of CD47 3′-UTR mutant F |  | CCTTTCCAGCTACTTTCGACTAATTCTATTTGTCTTCTC |  | *--* |
| Position 2504-2510 of CD47 3′-UTR mutant R |  | GAGAAGACAAATAGAATTAGTCGAAAGTAGCTGGAAAGG |  | *--* |

**Table S4.** SgRNA and primer sequences were used for CRISPR/Cas9-mediated miR-96-5p knockout and validation. (F, forward primer; R, reverse primer)

| Name (sgRNA or primer) | Primer sequence (5′-3′) | | |  | Restriction enzyme |
| --- | --- | --- | --- | --- | --- |
|  | Forward |  | Reverse |  |  |
| has-miR96-sgRNA1 | CACCGATCGGCCAAGCAGATGGCAC |  | AAACGTGCCATCTGCTTGGCCGATC |  | *BbsI* |
| has-miR96-sgRNA2 | CACCGAGTGCCAATATGGGAAAAGC |  | AAACGCTTTTCCCATATTGGCACTC |  | *BbsI* |
| has-miR96-sgRNA3 | CACCGCCGCACCAGTGCCATCTGCT |  | AAACAGCAGATGGCACTGGTGCGGC |  | *BbsI* |
| has-miR96-sgRNA4 | CACCGATCATGTGCAGTGCCAATAT |  | AAACATATTGGCACTGCACATGATC |  | *BbsI* |
| mmu-miR96-sgRNA1 | CACCGCTGTTCCAGTACCATCTGCT |  | AAACAGCAGATGGTACTGGAACAGC |  | *BbsI* |
| mmu-miR96-sgRNA2 | CACCGCGGCCACGTTCACCTCCCC |  | AAACGGGGAGGTGAACGTGGCCGC |  | *BbsI* |
| mmu-miR96-sgRNA3 | CACCGTACTGGAACAGGCCCTCTG |  | AAACCAGAGGGCCTGTTCCAGTAC |  | *BbsI* |
| mmu-miR96-sgRNA4 | CACCGATGGGAAAAGCGGGCTGCTG |  | AAACCAGCAGCCCGCTTTTCCCATC |  | *BbsI* |
| Scramble sgRNA | CACCGGCGAGGTATTCGGCTCCGCG |  | AAACCGCGGAGCCGAATACCTCGCC |  | *BbsI* |
| hsa-miR-96 | AGTGCTCCTAGACGTCGGAA |  | CCTCTTGGAGCTGTTCGGAG |  | *--* |
| mmu-miR-96 | GGCACTGGTAGAATTCACT |  | AACAGGGCATCACAGAAG |  | *--* |

**Supplementary Figures**


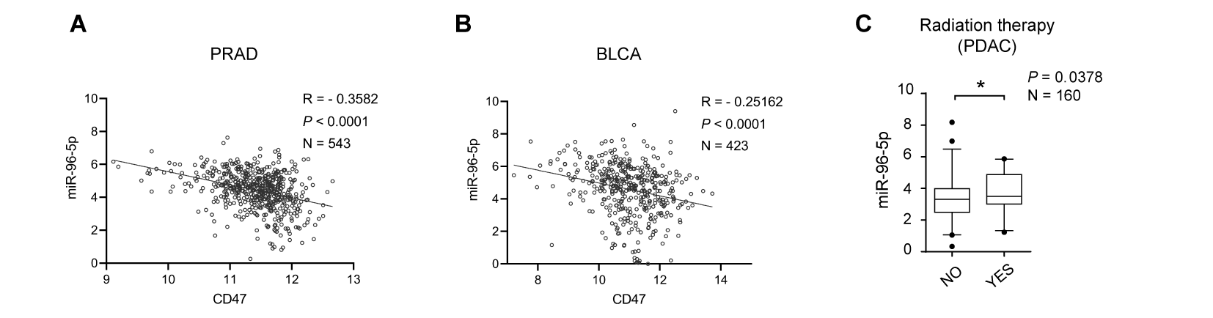


**Fig. S1.** **(A-B)** A correlation analysis between miR-96-5p and CD47 was conducted in PRAD (n = 543) and BLCA (n = 423) using data from TCGA. **(C)** The Wilcoxon test indicated a significant effect of radiation therapy on miR-96-5p levels in PDAC (P = 0.0378).


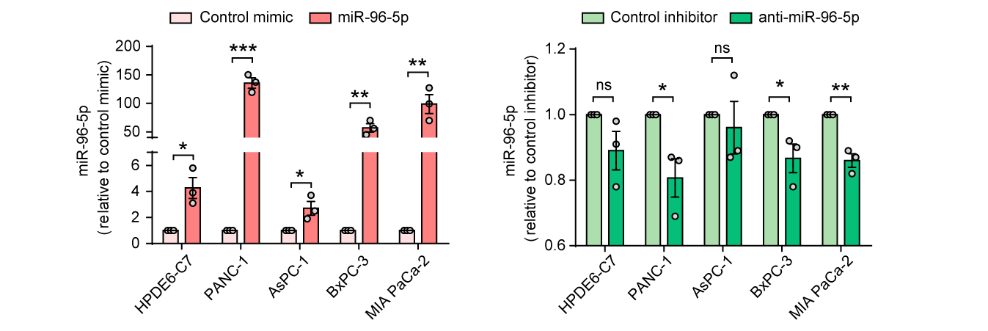


**Fig. S2.** qRT-PCR quantification of miR-96-5p after transfection with miR-96-5p mimics and inhibitors in various PDAC cells for 48 hours.


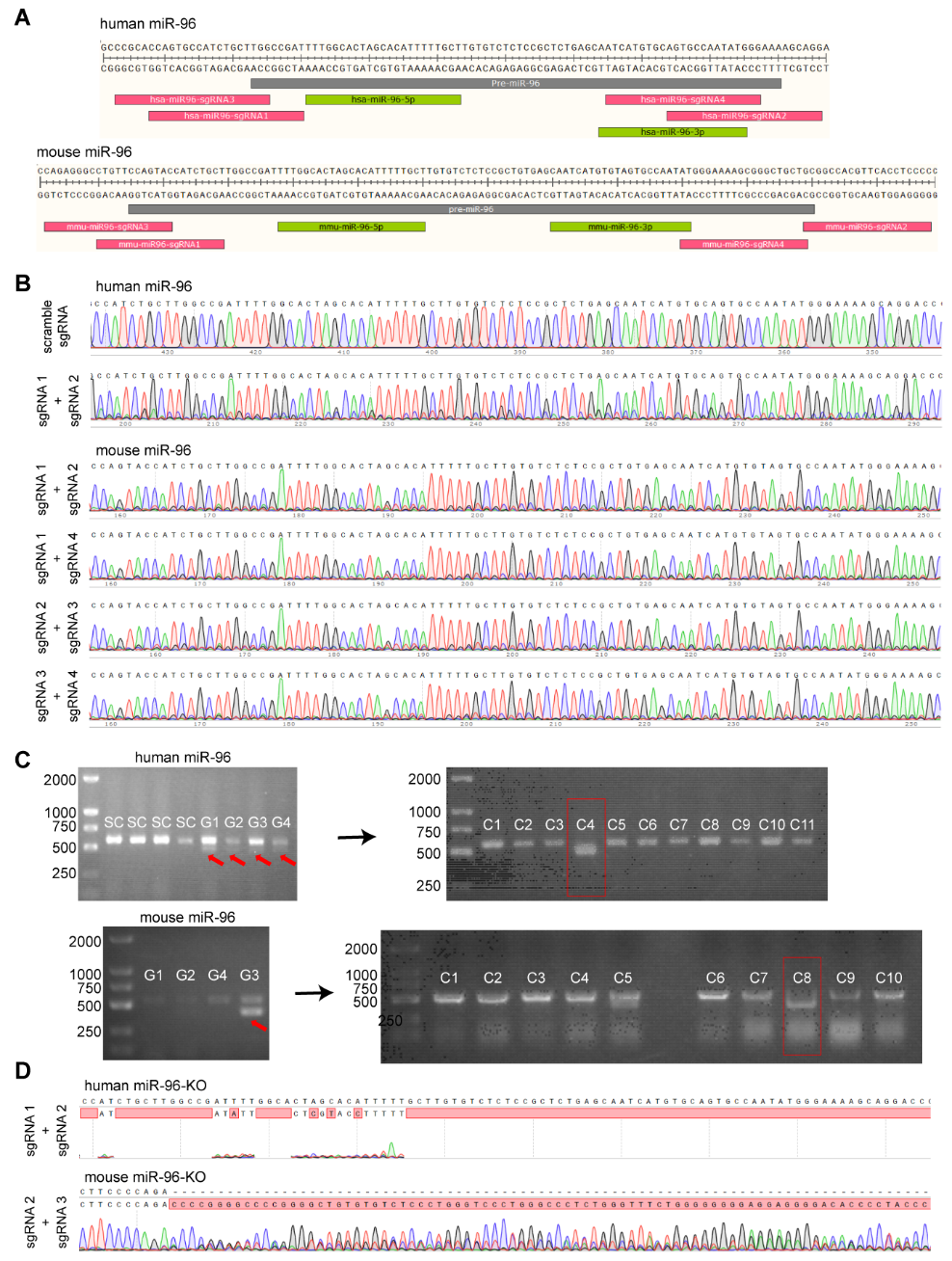


**Fig. S3.** Validation of CRISPR/Cas9-mediated miR-96-5p knockout. **(A)** SgRNAs that target both sides of human and mouse pre-miR-96. **(B)** Two sgRNA plasmids (Group 1, sgRNA1 and sgRNA2; Group 2, sgRNA1 and sgRNA4; Group 3, sgRNA3 and sgRNA2; Group 4, sgRNA3 and sgRNA4) were cotransfected into cells for 24 hours, followed by puromycin screening for 48 hours, then genomic DNA was extracted and specific target sequences were amplified for DNA sequencing. **(C)** After specific target sequences were amplified, PCR products were analyzed by electrophoresis on a 1.5% (w/v) agarose gel, and the transfection with two bands was selected for subsequent monoclonal screening. The red arrows indicate combinations that had better knockout efficiency. (SC: scramble sgRNA transfection; G1, cotransfection of sgRNA1 and sgRNA2; G2, cotransfection of sgRNA1 and sgRNA4; G3, cotransfection of sgRNA3 and sgRNA2; G4, cotransfection of sgRNA3 and sgRNA4). Right: identification of the positive clones by PCR. PCR products were electrophoresed on a 1.5% (w/v) agarose gel, and the sizes of the negative and positive clone fragments from human and mouse clones were about 680/600 bp and 540/430 bp, respectively. (Lanes C1-C11 or C1-C10: picked clones). **(D)** The sequencing results of positive clones were aligned with the genome sequence.


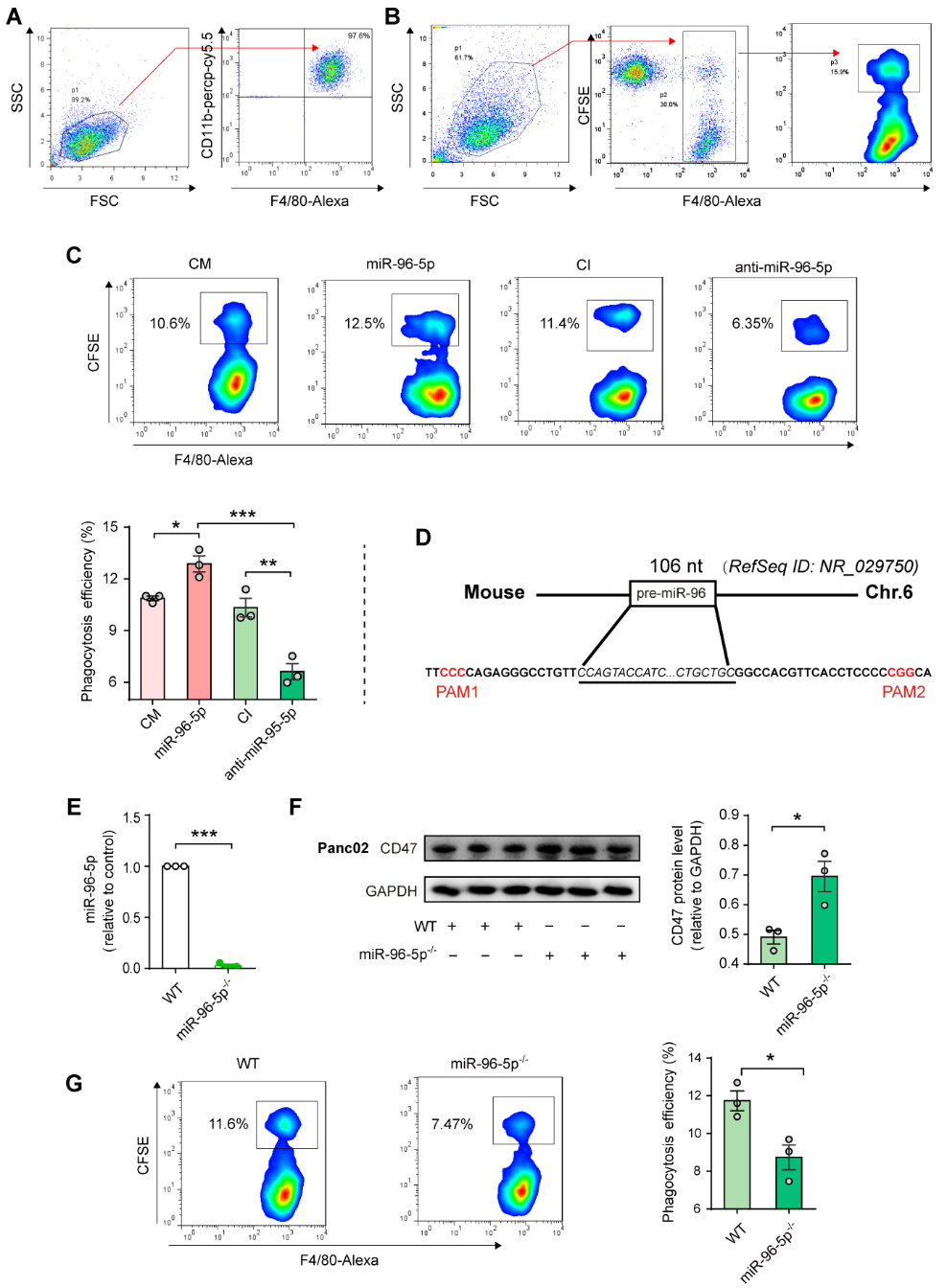


**Fig. S4** miR-96-5p increases phagocytosis of PDAC cells by mouse macrophages. **(A)** Flow cytometry analysis of macrophage markers CD11b and F4/80 after M-CSF induced murine bone marrow to differentiate into phagocytes. **(B)** The gating strategy applied in flow cytometry for the phagocytosis experiment. **(C)** After the overexpression or inhibition of miR-96-5p, the phagocytosis of BMDM-derived macrophages on Panc02 cells was analyzed by flow cytometry. **(D)** A diagram illustrating the deletion of pre-miR-96 mediated by the CRISPR/Cas9 system. Pre-miR-96 and PAM sequences are denoted by underlining and red-highlighting, respectively. **(E)** qRT-PCR analysis of the miR-96-5p levels in wild-type (WT) and miR-96-5p^-/-^ Panc02 cells. **(F)** Western blot analysis of the CD47 protein levels in WT and miR-96-5p^-/-^ Panc02 cells. **(G)** Phagocytosis of WT and miR-96-5p^-/-^ Panc02 cells by BM-derived macrophages was determined by flow cytometry.


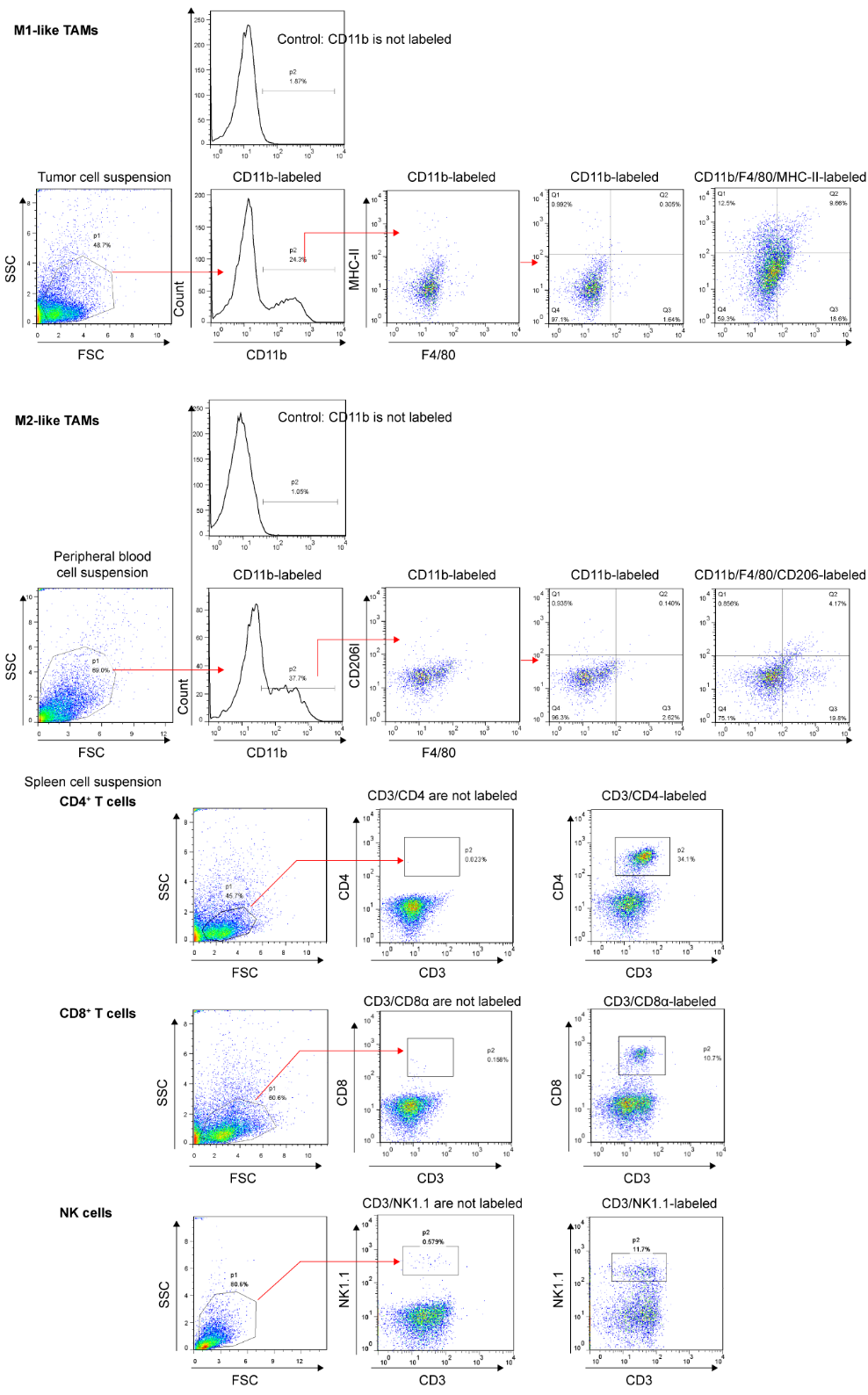


**Fig. S5** Gating strategies in flow cytometry were applied to evaluate the proportions of M1, M2, CD4, CD8, and NK cells.


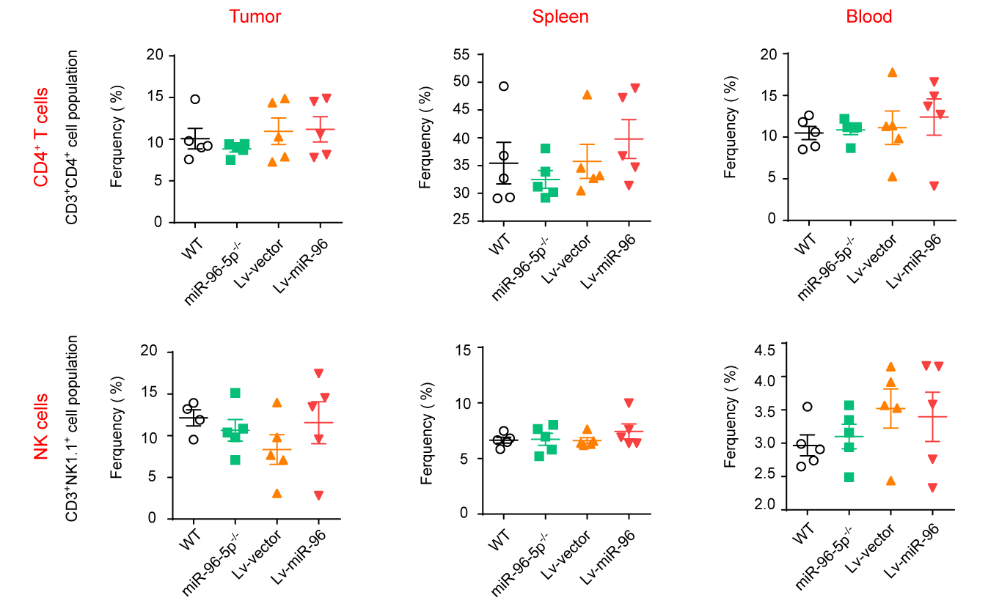


**Fig. S6** Effects of miR-96-5p on CD4^+^ T and NK cells in the tumors, spleen, and blood of mice bearing Panc02 cells with wild-type, miR-96-5^p-/-^, Lv-vector, and Lv-miR-96.


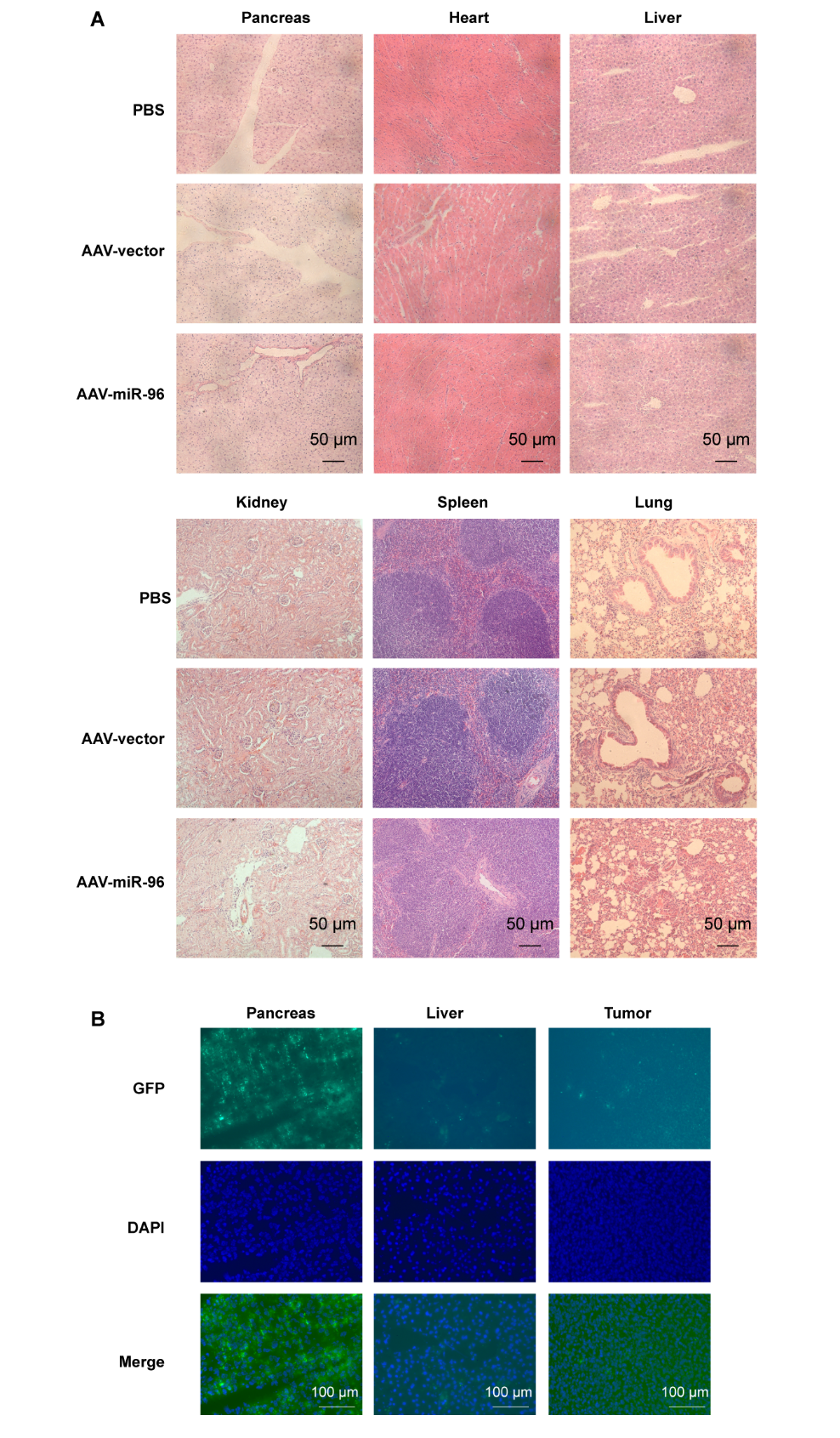


**Fig. S7** The morphologic effect and targeting analysis of AAV-PAN virus injection on various tissues and organs in mice. (A) HE detection of the effects of AAV-mediated high expression of miR-96-5p on the morphology of mouse tissues and organs. (B) GFP expression in pancreas, pancreatic tumors, and liver tissues was detected by fluorescence microscopy.


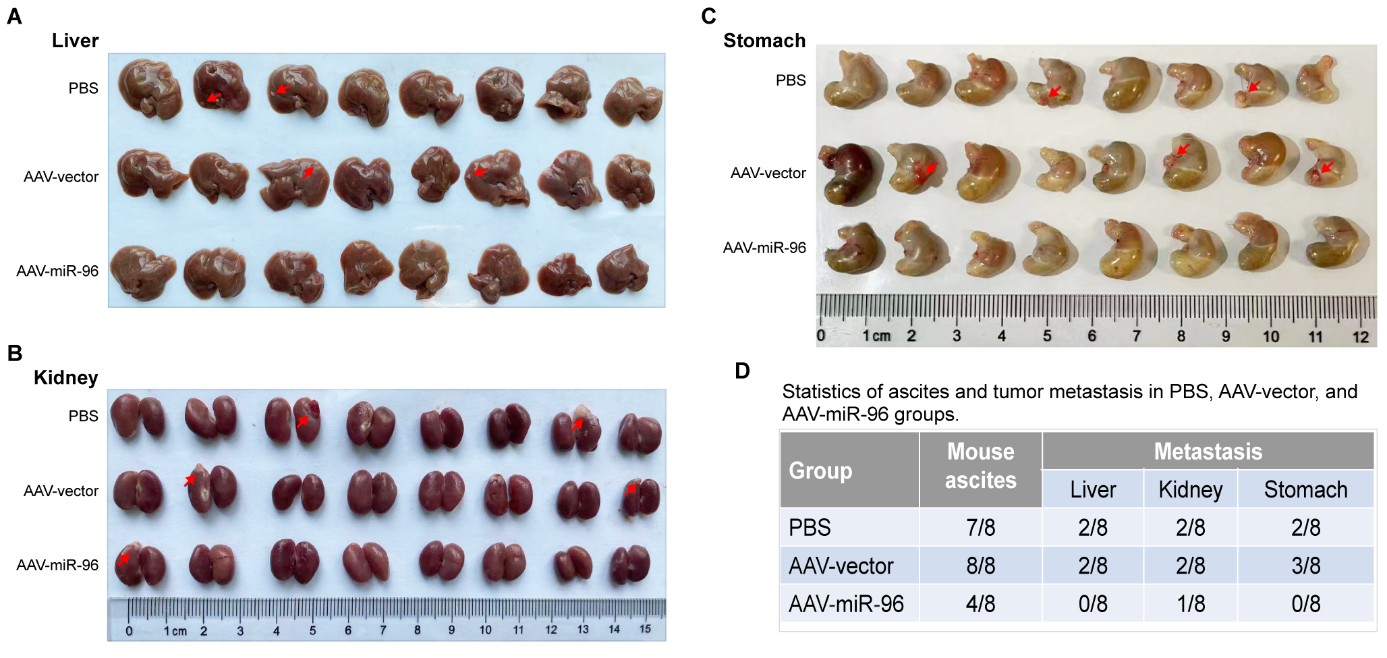


**Fig. S8** Statistical analysis of ascites and pancreatic tumor metastasis in the liver, kidney, and stomach of mice.
