# Supplementary material for: miR-96-5p-mediated Inhibition of CD47 contributes to pancreatic tumor regression via activating both innate and adaptive anti-tumor immunity
Source: Cell Commun Signal. 2025 Dec 5;24:16. doi: 10.1186/s12964-025-02582-5 (PMC12797448; doi:10.1186/s12964-025-02582-5)

Original image for Fig. 2C


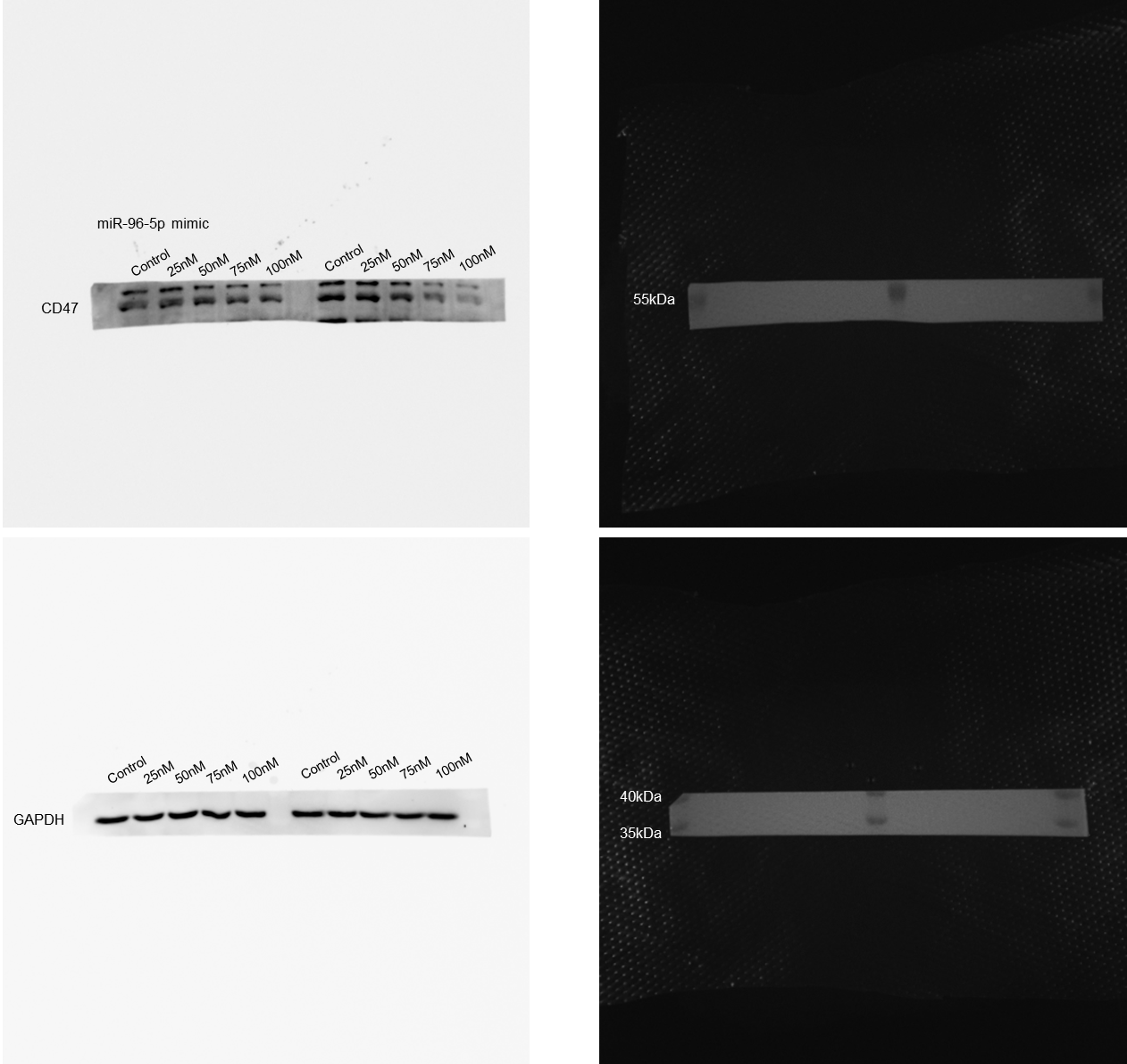


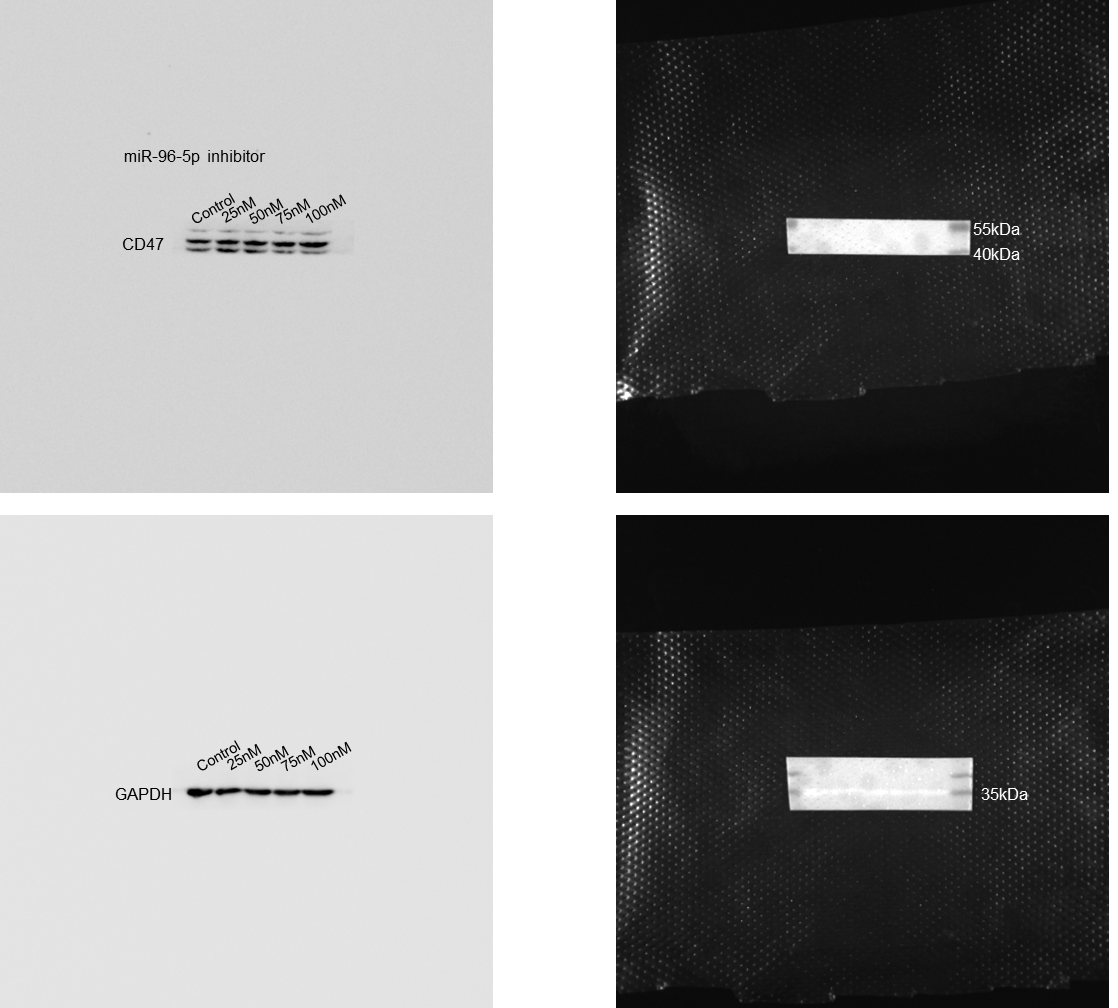


Original image for Fig. 2D


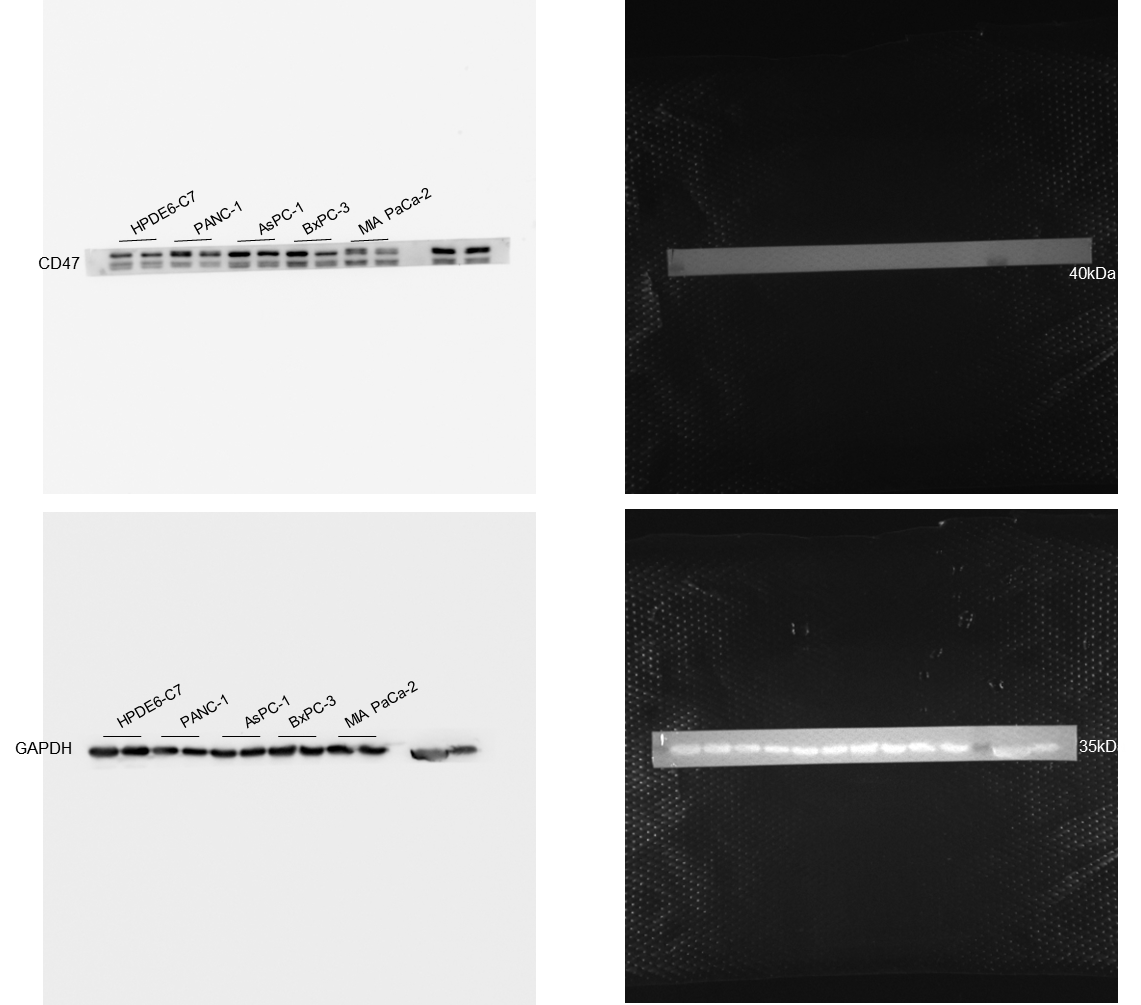


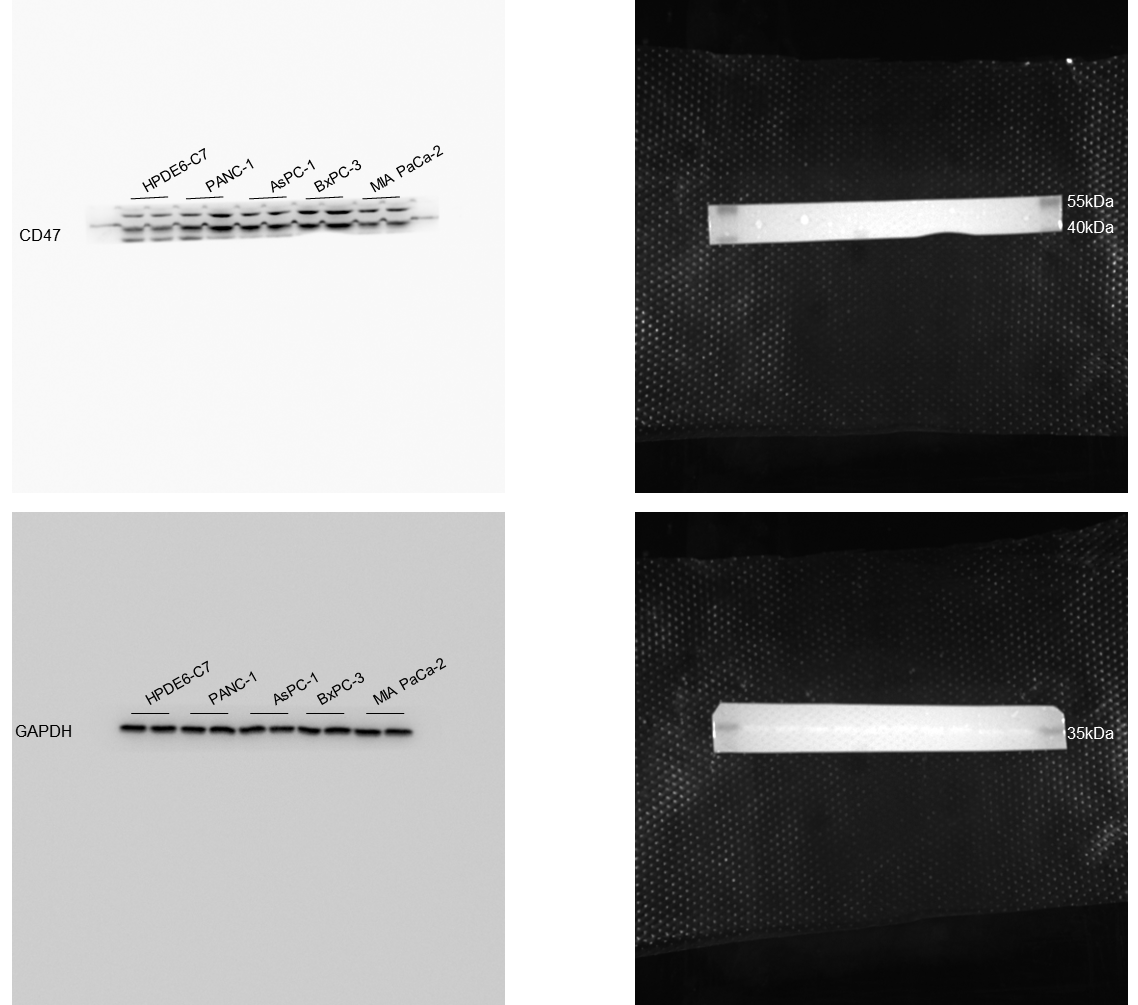


Original image for Fig. 2K


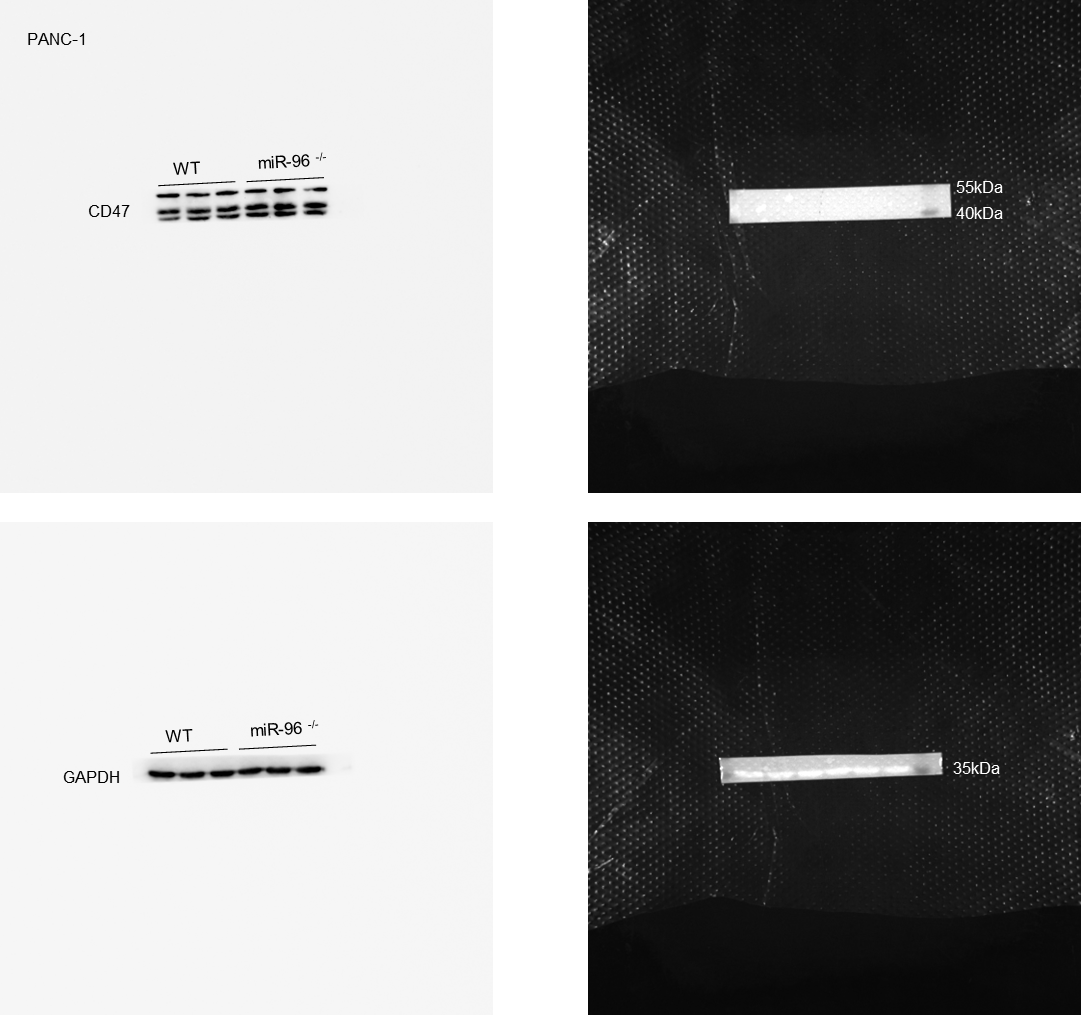


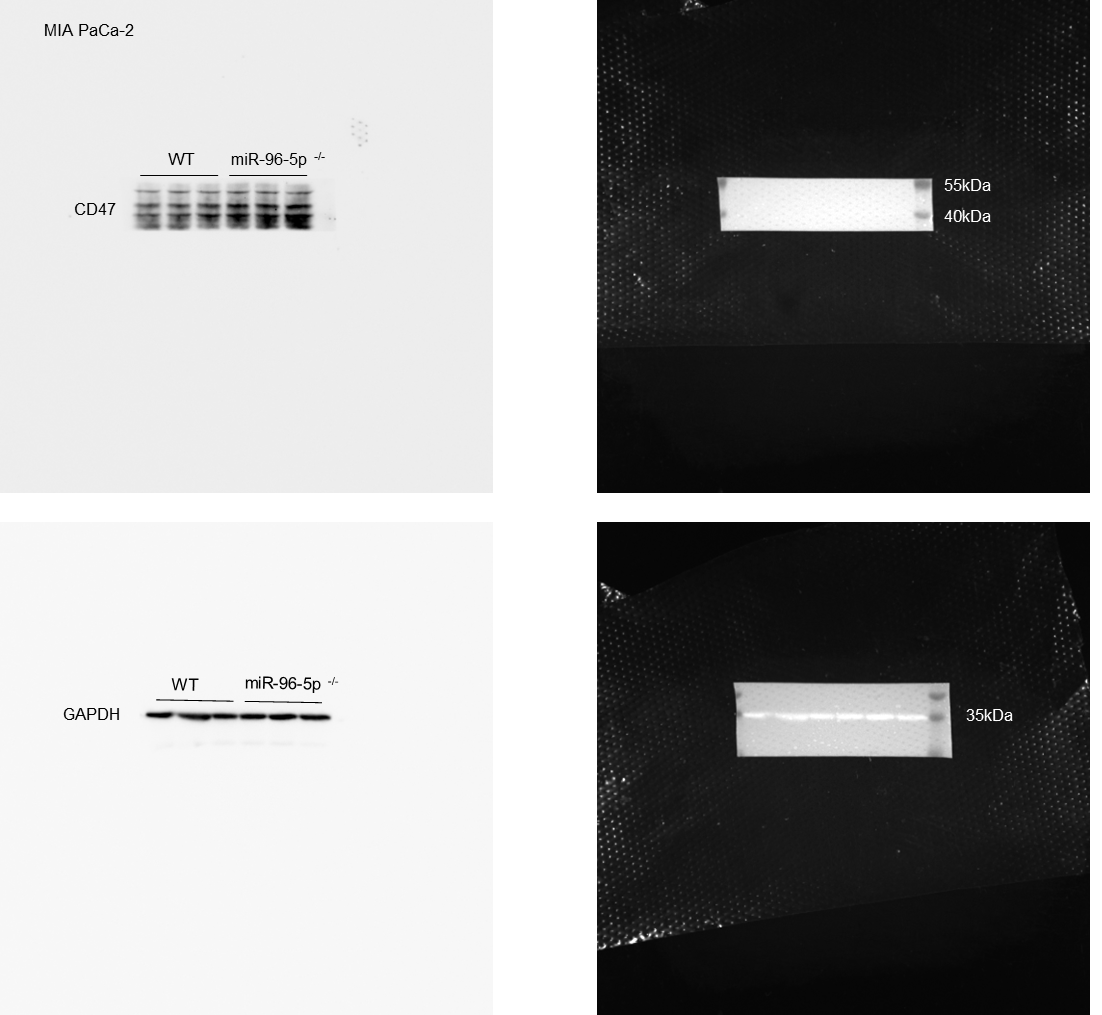


Original image for Fig. 3E


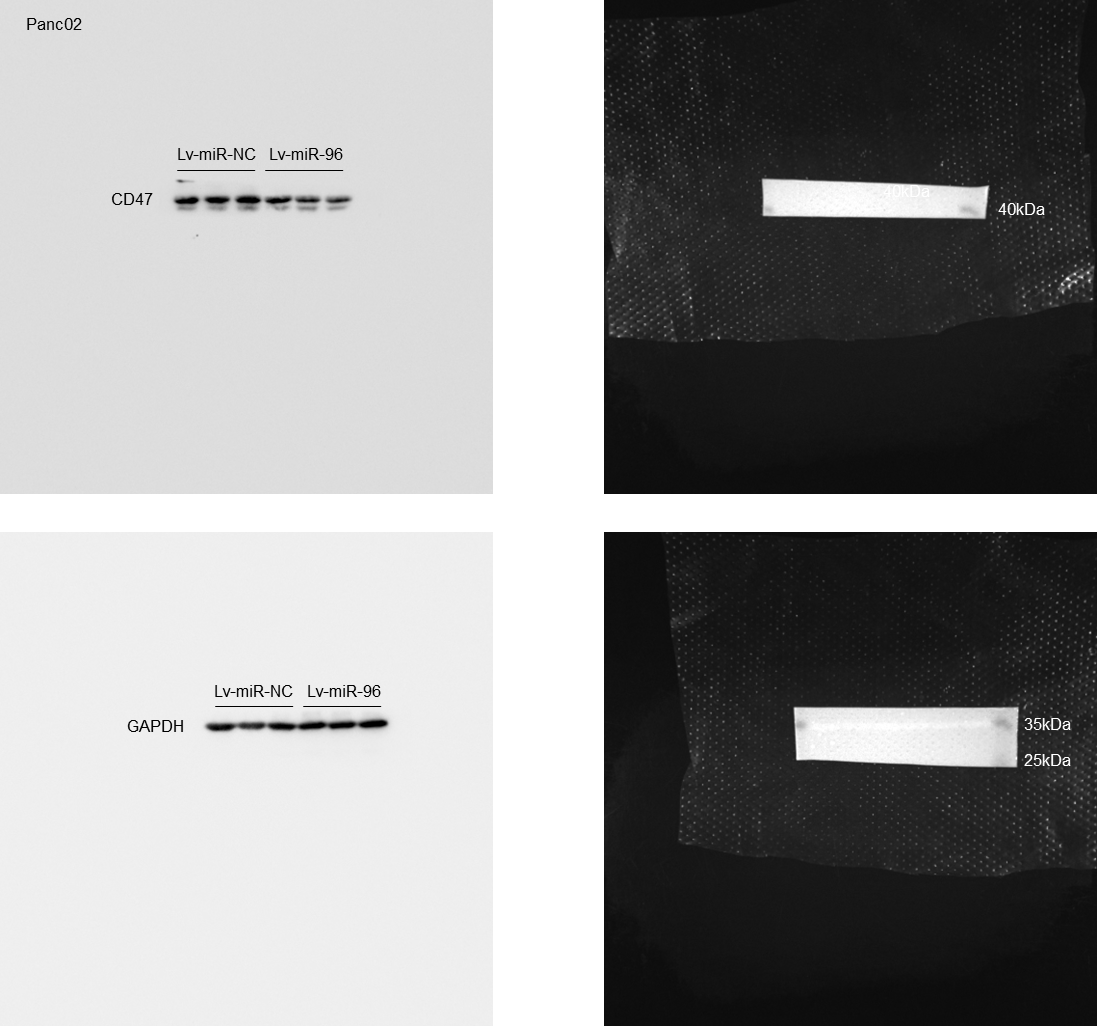


Original image for Fig. 3J


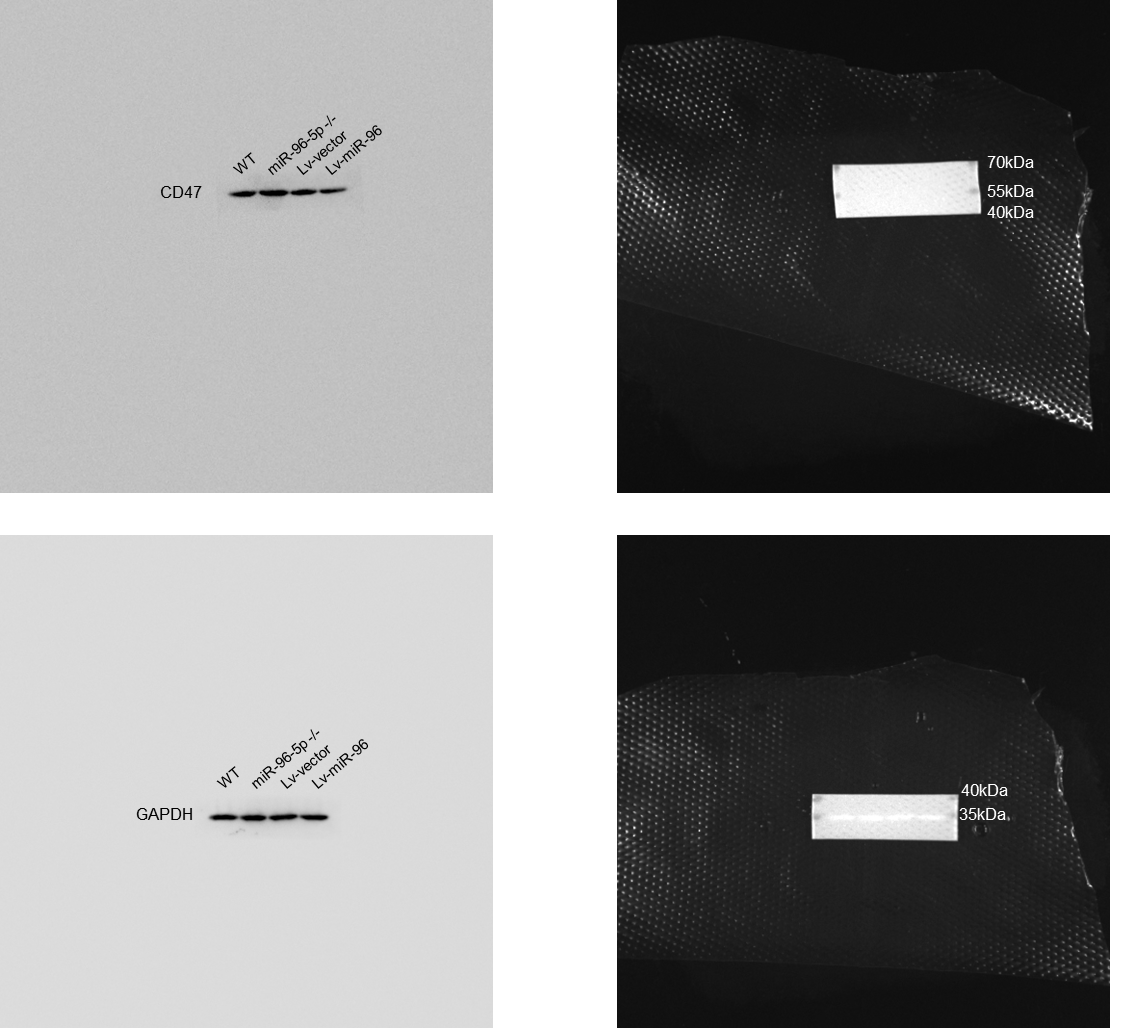


Original image for Fig. 4F


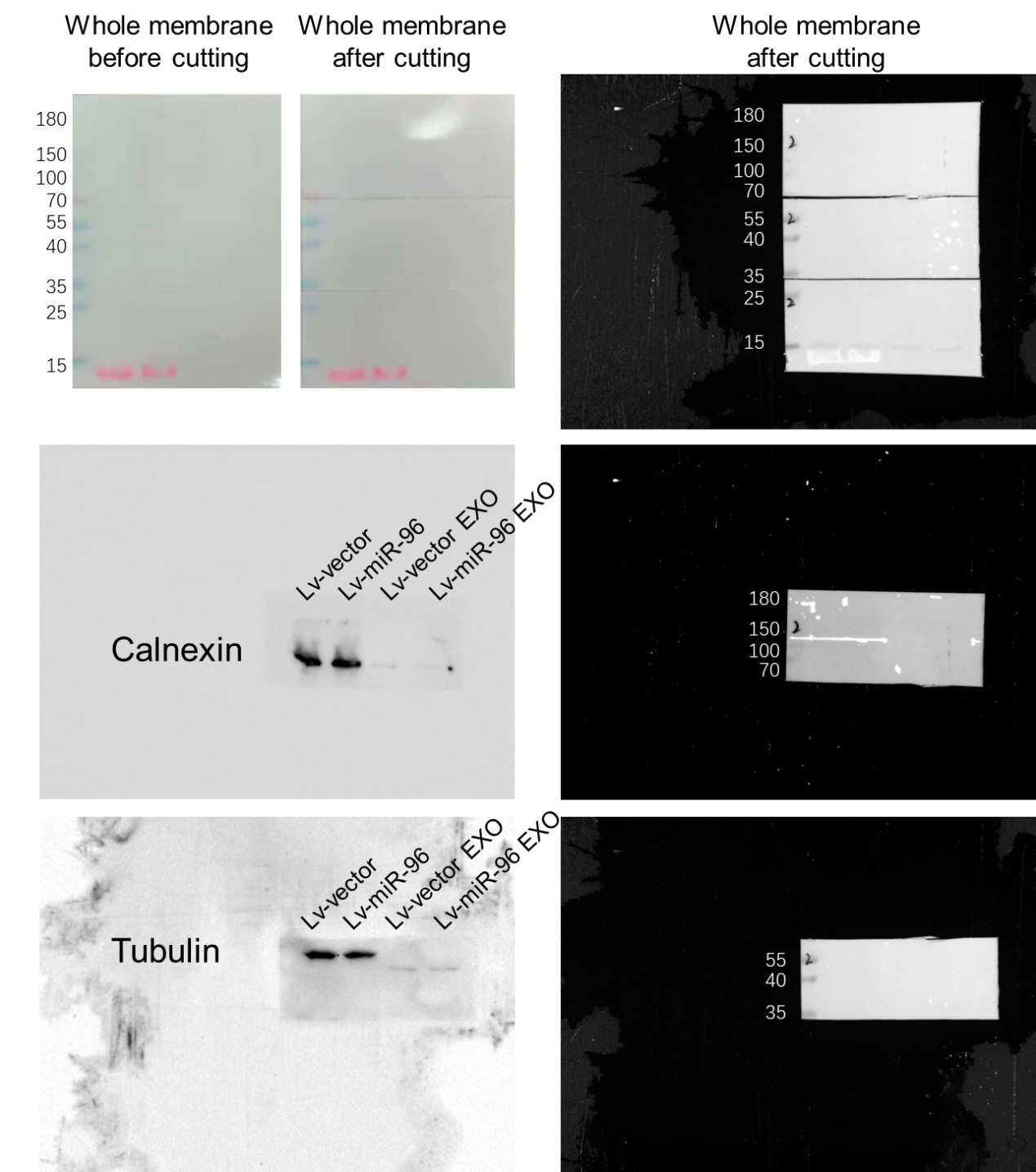


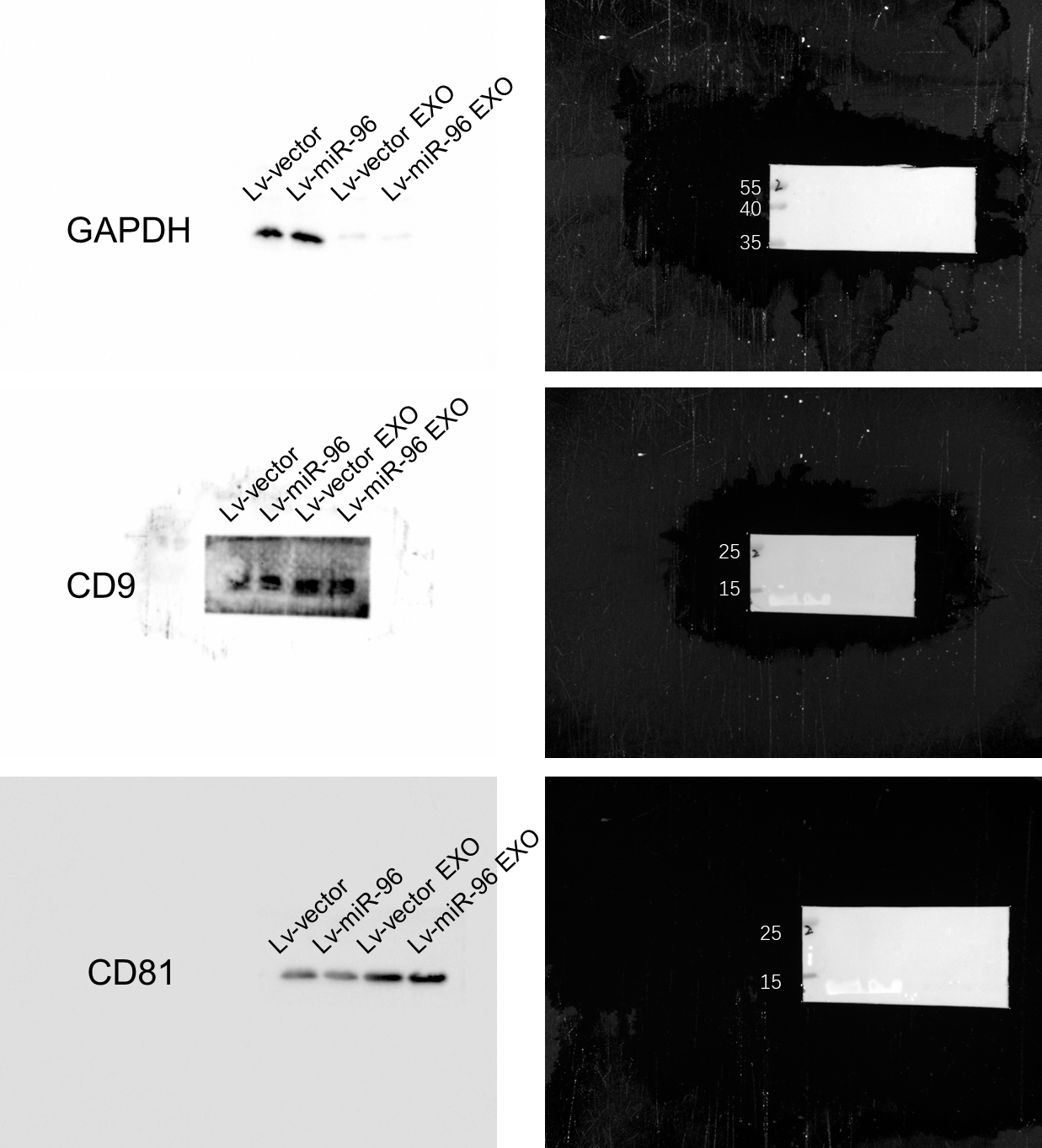


Original image for Fig. 5B


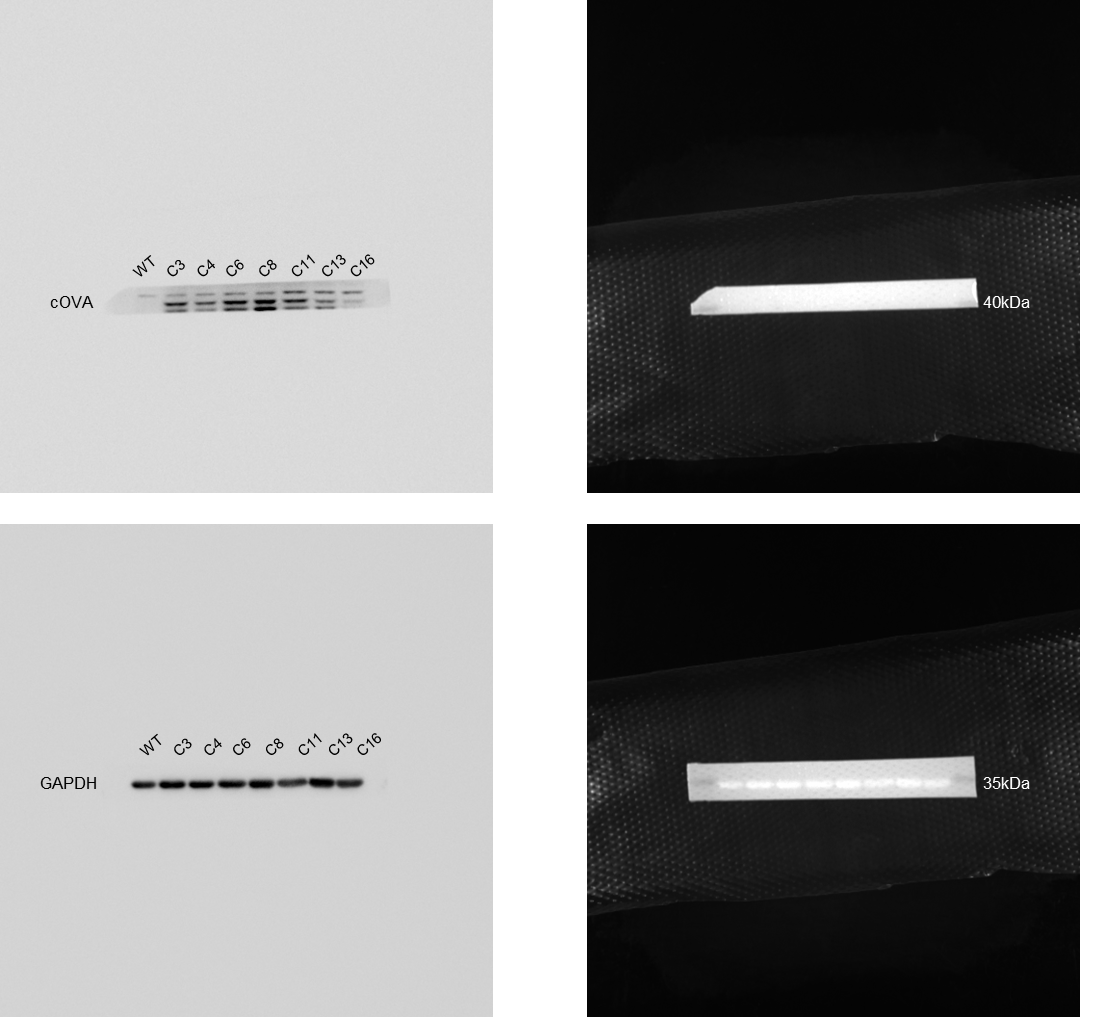


Original image for Fig. 7H


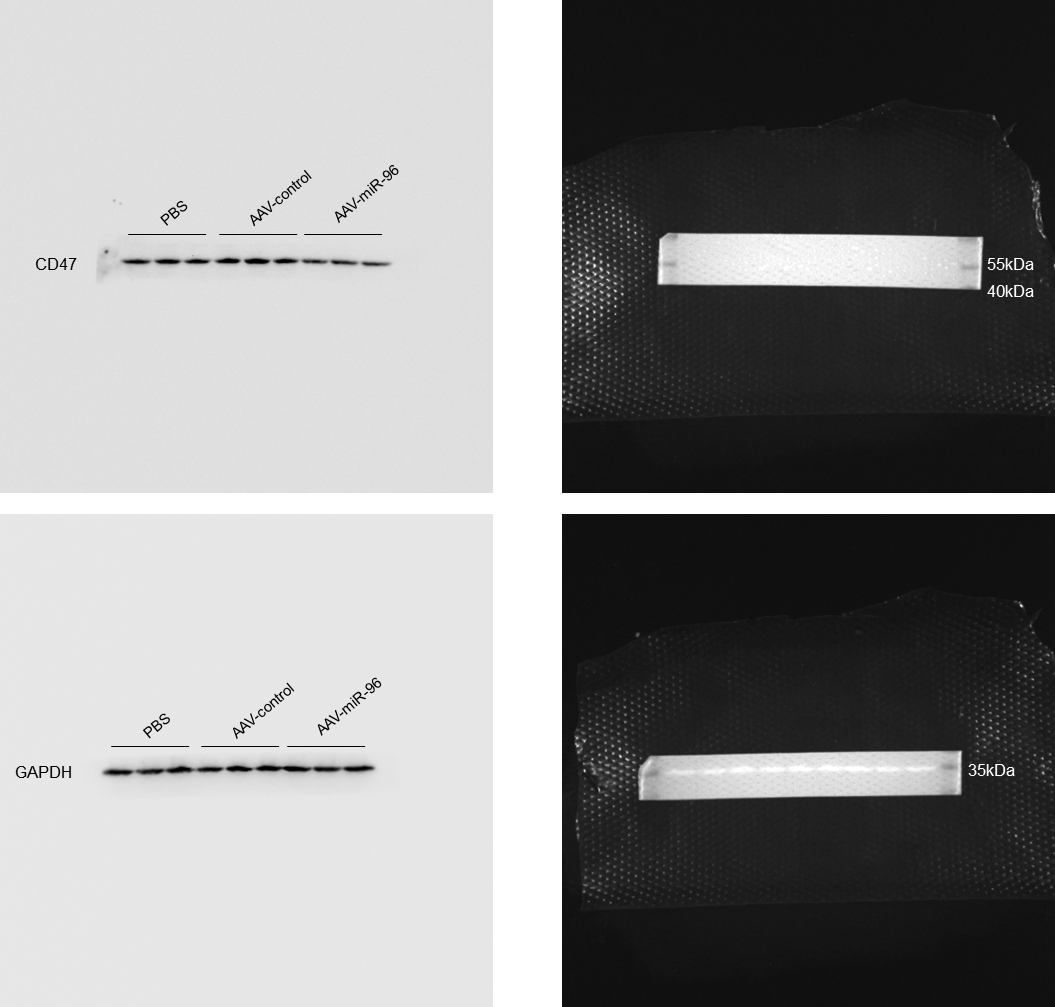

Supplement: Supplementary file 2 — Supplementary material 2. [file 12964_2025_2582_MOESM2_ESM.docx]
